# Conservation priorities for endangered Indian tigers through a genomic lens

**DOI:** 10.1038/s41598-017-09748-3

**Published:** 2017-08-29

**Authors:** Meghana Natesh, Goutham Atla, Parag Nigam, Yadvendradev V. Jhala, Arun Zachariah, Udayan Borthakur, Uma Ramakrishnan

**Affiliations:** 1 National Center for Biological Sciences, Tata Institute of Fundamental Research, Bangalore, 560065 India; 2 Shanmugha Arts, Science, Technology and Research Academy (SASTRA) University, Tirumalaisamudram, Thanjavur, 613401 Tamil Nadu India; 3 Wildlife Institute of India, Chandrabani, Dehradun, 248001 India; 4 Kerala Veterinary and Animal Sciences University, Lakkidi Post, Pookode, Kerala 673576 India; 5 Aaranyak, 12 Kanaklata Path in Lachit Path, Ajanta Path, Survey, Beltola, Guwahati, 781028 Assam India

## Abstract

Tigers have lost 93% of their historical range worldwide. India plays a vital role in the conservation of tigers since nearly 60% of all wild tigers are currently found here. However, as protected areas are small (<300 km^2^ on average), with only a few individuals in each, many of them may not be independently viable. It is thus important to identify and conserve genetically connected populations, as well as to maintain connectivity within them. We collected samples from wild tigers (*Panthera tigris tigris*) across India and used genome-wide SNPs to infer genetic connectivity. We genotyped 10,184 SNPs from 38 individuals across 17 protected areas and identified three genetically distinct clusters (corresponding to northwest, southern and central India). The northwest cluster was isolated with low variation and high relatedness. The geographically large central cluster included tigers from central, northeastern and northern India, and had the highest variation. Most genetic diversity (62%) was shared among clusters, while unique variation was highest in the central cluster (8.5%) and lowest in the northwestern one (2%). We did not detect signatures of differential selection or local adaptation. We highlight that the northwest population requires conservation attention to ensure persistence of these tigers.

## Introduction

Genetic variation and its partitioning across populations helps inform evolutionary history, population connectivity, and demographic fluctuations^1, 2^. Such information is particularly important for endangered species, where conservation strategy and management planning depends on accurate detection of species status, population structure and connectivity, inbreeding, adaptive variation, admixture, and population demographic history^2^.

Until recently, highly polymorphic microsatellites  were the markers of choice for conservation genetic studies^3^. Rapid development of next generation sequencing (NGS) has facilitated survey of genome-wide variation. Such data has helped identify African savannah (*Loxodonta africana*) and forest elephants (*Loxodonta cyclotis*) as separate species^4^, detect cryptic population structure in chimpanzees (*Pan troglodytes*)^5^, reveal local adaptation in giant panda (*Ailuropoda melanoleuca*) populations^6^ and quantify genetic diversity in Tasmanian devils^7^ (*Sarcophilus harrisii*). Genome-wide Single Nucleotide Polymorhphisms (SNPs) have been developed for monitoring populations in the wild, including bears^8^ (*Ursus arctos*), wolves^9^ (*Canis lupus*) and river otters^10^ (*Lontra canadensis*). Several thousands of SNPs provide higher statistical power in comparison to examining a limited number of microsatellites, resulting in more robust population genetic inference^11, 12^.

Many large carnivores are currently threatened, with around 61% of them designated as ‘threatened’^13^ by the IUCN redlist. A majority of them have suffered range reduction and fragmentation, with surviving individuals restricted to small isolated populations that tend to have low genetic variation and a high probability of extinction^14^. Identifying such populations can help devise conservation strategies to avoid inbreeding depression, aid in population recovery and plan assisted gene flow for genetic rescue. Such strategies have been implemented effectively in carnivores like the Florida panther^15^ (*Puma concolor coryi*). However, genetic rescue could be counter-productive if populations are locally adapted^16^. Conservation strategy must consider the relative risks of inbreeding and outbreeding depression before genetic rescue is implemented^17^.

Tigers have lost an estimated 93% of their historical range^18^. Of the nine tiger subspecies, four are extinct (*P. tigris sondaica*, *P. Tigris balica*, *P. tigris virgata*, and *P. tigris amoyensis*). The remaining five subspecies have experienced intense population bottlenecks (e.g. 90% decline in the Indo-Chinese tigers)^19, 20^ in historical times and prolonged reduction in distribution^21^. Tiger conservation and recovery is a global priority as exemplified by several international efforts (e.g. Global Tiger Forum). Recent surveys suggest an increase in tiger numbers^22^ indicating the success of such initiatives.

The Indian subcontinent is home to more than 60% of all tigers and harbors over half the global genetic diversity of the species^23^. Securing the future of Indian tigers is critical for survival of the species. From a time when tigers were widespread^24^, today tigers survive in small (median = 19, mean = 35)^25^, often isolated populations, many of which may not be viable on their own. Tiger monitoring is conducted at the scale of protected areas or geographically defined landscapes (e.g., central India) with clustered protected areas. Data on historical occupancy of Indian tigers^24^ reveals relatively continuous distributions. This implies that tiger genetic clusters may have wider geographical extents than currently monitored landscape units, as supported by microsatellite data^20^. However, tiger monitoring surveys indicate that tiger populations within some geographic landscapes have become isolated and habitat connectivity between others is tenuous^25^. Additionally, these landscapes represent different biogeographic zones, such as the Semi-Arid or the Western Ghats, with distinctive habitats^26^ such as grasslands, subtropical moist forests, mangroves and tropical dry forests^27^. Given their wide distribution, tigers may be categorized as generalist species. However, tiger populations living in different habitats may potentially be locally adapted; in such cases genetic rescue may not be considered a valid conservation strategy^17^. Therefore, understanding the spatial extent of genetic clusters as determined by genetic connectivity and local adaptation is essential for conservation.

In this paper, we collect genome-wide SNP data from wild tigers to better understand how protected area-based populations in India are segregated into genetic clusters. We investigate the following questions: (i) what are the genetic clusters for tigers in India? (ii) how is genetic variation distributed among these genetic clusters? (iii) are there any signatures of differential local adaptation between these clusters?

## Results

### Sequencing Data, SNP calling, and Filtration

The total number of retained reads per sample varied from 4,869 to 12,882,482, with an average of 4,118,660 (as seen in the output of process_radtags command from the program Stacks^28^). After alignment, the program Stacks was used to assess the depth at unique loci obtained per individual. The number of unique loci (as called by Stacks) obtained per sample (Table S2) was also highly variable, ranging from 1 to 693,476, with an average of 268,964. The raw vcf file (after calling SNPs in Freebayes^29^) had 1,527,595 loci. This vcf file was passed through several filters, as mentioned in the methods. In the final dataset, only individuals with less than 25% missing data (although the average percentage of missing data was much lower at 4.2%) and SNPs with less than 5% missing data were retained. Details of samples removed are presented in Table S3.

### Genetic Differentiation

**Figure 1:**
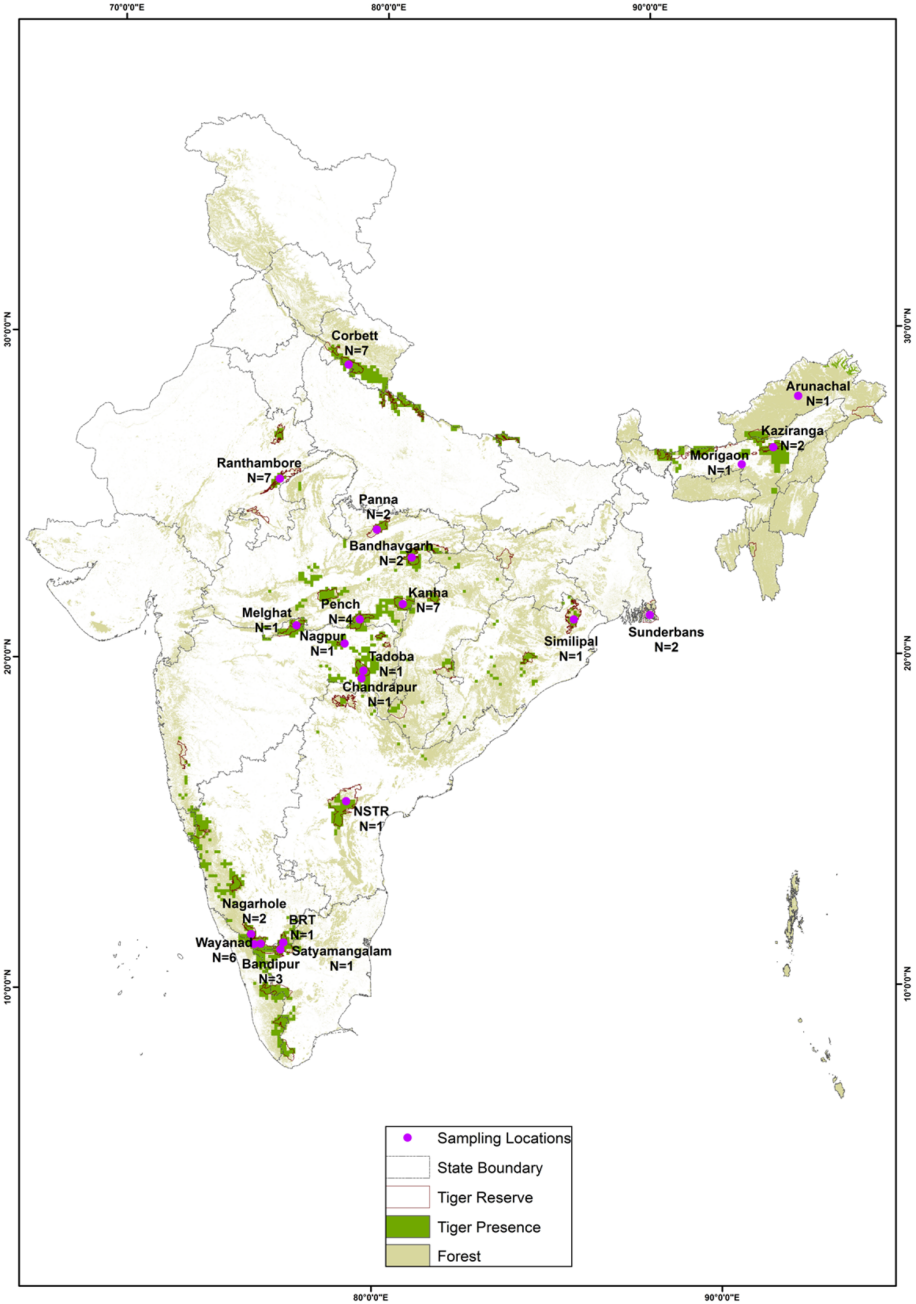
Map depicting tiger reserves in India. The total number of samples obtained for the study was 54 from across 21 locations (Table S1). Sample size from each location is denoted by ‘N’. Of these samples however, only 38 were used for the final analyses. The map was generated using the ArcGIS software (Desktop), an ESRI product version 10.2. (http://www.esri.com/software/arcgis/arcgis-for-desktop). The forest cover map, published in the State of Forest report-2013 (http://fsi.nic.in/cover_2013/sfr_forest_cover.pdf), was purchased from the Forest Survey of India, and the Protected Area boundaries from the Wildlife Institute of India, Dehradun.

**Figure 2:**
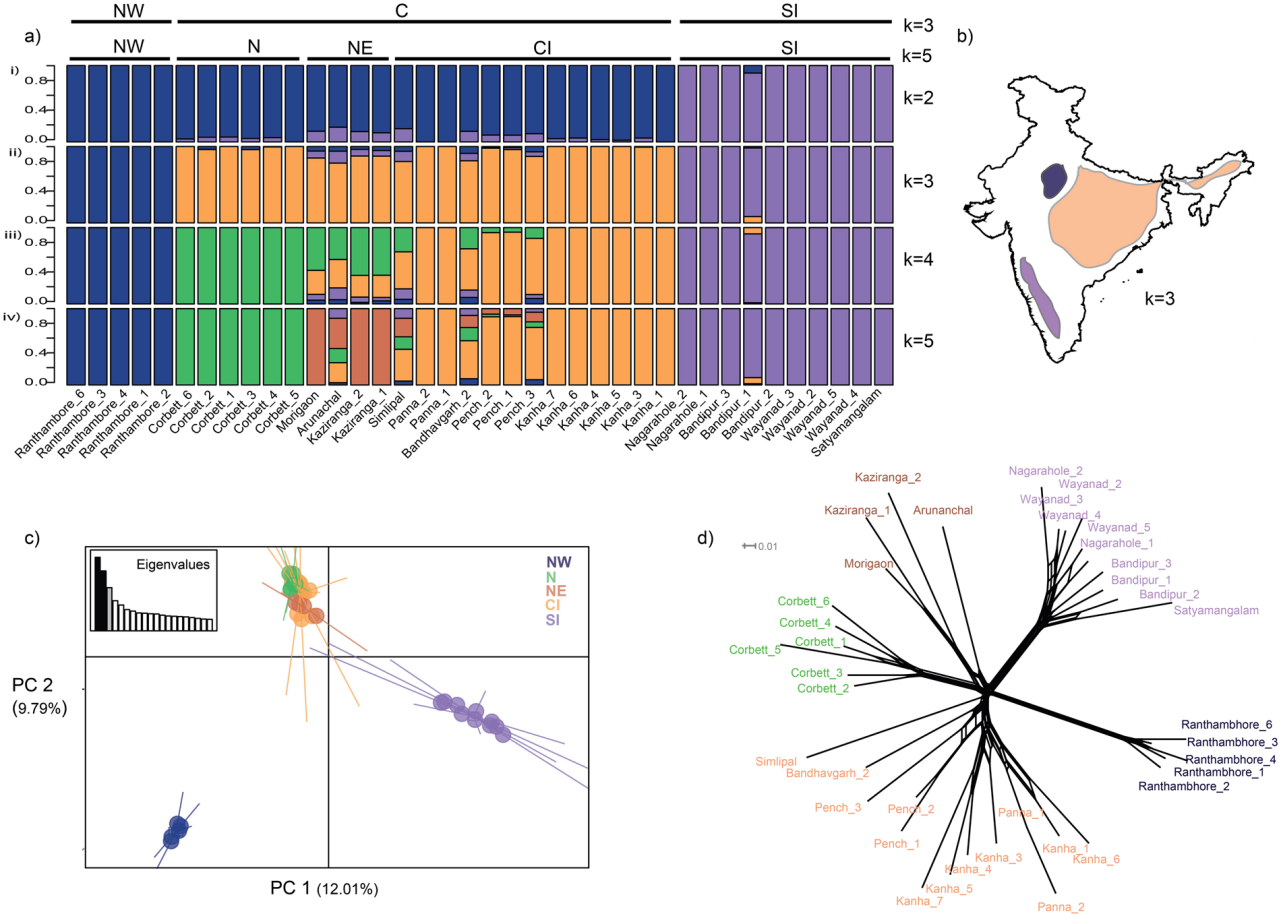
Genetic clusters inferred using multiple methods. (**a**) Genetic clusters inferred at different values of K (2–5) in Admixture. Each vertical bar indicates a single individual, with the Y axis depicting the proportion derived from each cluster. The optimal suggested value of number of clusters was K = 3. The cluster names above indicate clusters detected at K = 3, and K = 5. (**b**) An illustration to depict the geographical extant of the three clusters inferred at K = 3. The figure was generated in QGIS version 2.0.1. (https://qgis.org/downloads/). The polygons have been added just for illustrative purposes. (**c**) Principal components analysis depicting the first and second principal components. The percentage of variation explained is given in brackets. (**d**) A phylogenetic network constructed in SplitsTree4. Sample names have been coloured for the purpose of representation only.

Genetic differentiation was assessed using 10,184 SNPs from 38 samples. Tigers from across India (Fig. 1) were partitioned into three major clusters in all analyses. Further sub-structuring within one cluster was indicated by most analyses. The three clusters identified by Admixture (best support for K = 3) and PCA (Fig. 2a,c), geographically coincided with individuals in the northwest, central and southern India. The first cluster, designated as the ‘NW’ cluster, was solely composed of individuals from a single protected area in the northwest region - Ranthambore (Fig. 1). The third cluster, designated ‘SI’, was comprised of all samples from southern India (Fig. 1). The remaining individuals formed a single cluster designated as ‘C’. Within this cluster, northeast samples (Arunachal, Kaziranga and Morigaon), Bandhavgarh and Simlipal derived between 12 and 21% of their ancestry from the other two clusters.

**Figure 3:**
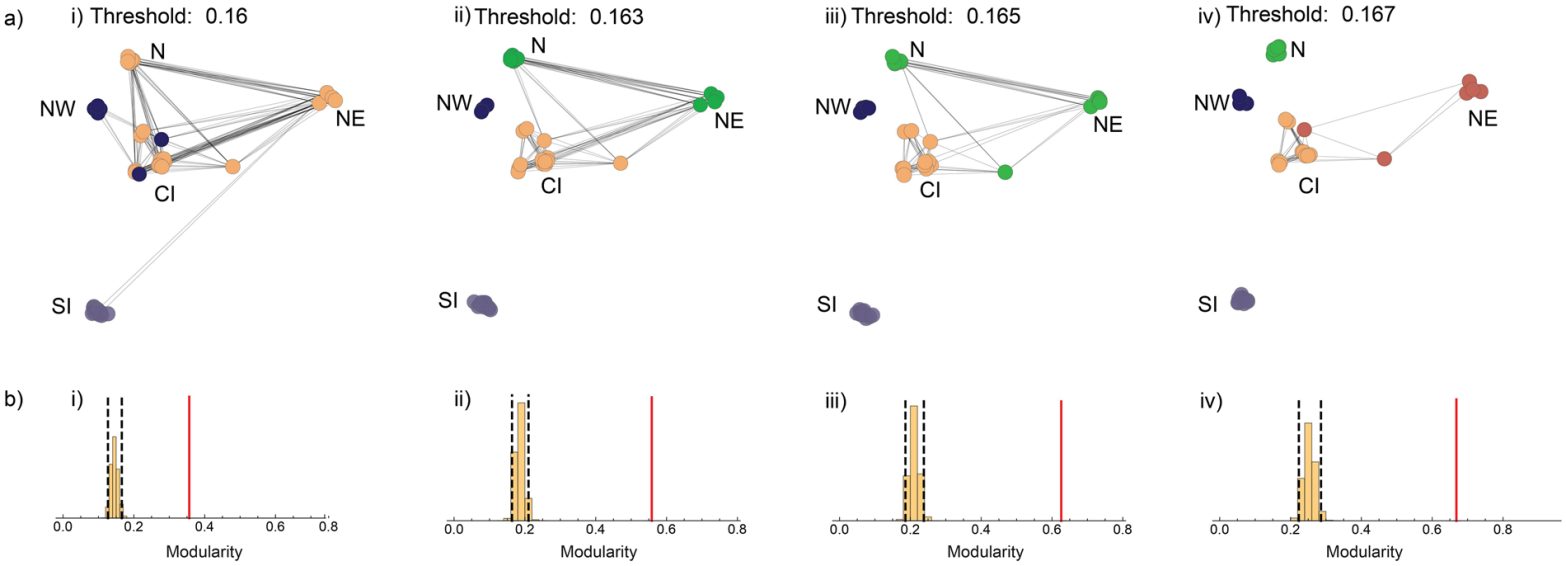
Networks plotted at four thresholds of genetic similarity. (**a**) Each panel (i–iv) in the figure depicts the threshold of genetic similarity used to connect individuals – 0.160–0.167, With increasing thresholds, dissimilar populations break off into separate modules. NW and SI separate into modules at a low threshold. Differentiation within C is evident at higher thresholds. Individuals are geographically placed. (**b**) Test of significance of modularity at each genetic similarity threshold. 5000 permutations were done to generate the distribution of the permuted data. The red line indicates the observed modularity.

Cluster C was further subdivided into ‘N’ (comprising individuals from the North), ‘NE’ (northeast individuals from Kaziranga, Morigaon and Arunachal) and ‘CI’ (remaining individuals from central India), supported to varying degrees by different analyses. The phylogenetic network (Fig. 2d) showed two further splits in the C cluster separating out N and NE, although these were shallower splits in comparison to SI and NW. Network analysis also supported the presence of hierarchical structure (Fig. 3a). At a low threshold of genetic similarity (0.16), three clusters were inferred, consistent with our clustering analyses. However, two individuals from central India were assigned to the NW cluster (albeit with very low affinity – Fig. S11). Community detection at higher thresholds (0.163, 0.165), identified four clusters, splitting C into a north- northeast (N-NE) and a CI cluster. A further increase in threshold (0.167) separated individuals from the northeast, Bandhavgarh and Simlipal into a fifth cluster. Admixture results for higher values of K (Fig. 2a) also supported this inference. Estimates of ancestry coefficients (range: 0.00001–0.99998, at K = 3) are provided in supplementary materials (Table S6).

### Summary Statistics

Theta (H) was highest for C (0.48), intermediate for SI (0.35) and lowest for NW (0.22) (Table 1). Mean heterozygosity (He, Table 1) followed a similar trend. Inbreeding coefficients (Fig. S5) were higher in NW (mean = 0.48) compared to other clusters. Pair-wise relatedness estimates (Supplementary Fig. S4) revealed two samples from Ranthambore were identical (pair-wise relatedness = 0.9) and hence one of these individuals was removed from all analyses. Overall, relatedness was high in NW as well (range = 0–0.5, mean = 0.27) compared to those in other protected areas, e.g. Kanha (0) and Wayanad (range = 0–0.45, mean = 0.19), and other genetic clusters (C: range = 0–0.65, mean = 0.08).

**Table 1:** Summary Statistics.

	NW	C	SI
Theta (H)	0.22	0.48	0.35
Mean H_e_ (sd)	0.18 (0.22)	0.33 (0.16)	0.26 (0.2)
Mean H_o_^#^(sd)	0.38 (0.22)	0.25 (0.15)	0.33 (0.19)

The table summarizes summary statistics (He, Ho, Pair-wise F_ST_ and Theta (H)) for the three main population clusters inferred at K = 3. ^#^These values are estimated based only on the number of polymorphic loci in each cluster (NW = 4482, C = 9601, SI = 7272).

Pair-wise F_ST_, estimated assuming three clusters (NW, C, SI), was highest for the NW–SI comparison (0.35, Table 2). When assuming five genetic clusters (NW, N, NE, CI and SI), with N, NE and CI combined as a group (Table 3), AMOVA revealed that 65.32% (V_d_) of the total variation observed was due to variation within individuals (p value = 0). Variation contributed by differences among groups (V_a_), although not statistically significant (p value = 0.09), was much lower (9.26%). F_IS_ was higher in CI and NE clusters (Table 4).

**Table 2:** Pair-wise F_ST_ comparisons.

	NW	C	SI
NW	0		
C	0.24*	0	
SI	0.35*	0.16*	0

(*) indicates values are significant at p < 0.05.

**Table 3:** AMOVA (assuming 5 populations; N, NE, CI one group).

	variation (%)	F statistics		p
Among groups (V_a_)	9.26	F_CT_	0.09	0.09
Among populations within groups (V_b_)	16.48	F_SC_	0.18	0
Among individuals within populations (V_c_)	8.94	F_IS_	0.12	0
Within individuals (V_d_)	65.32	F_IT_	0.35	0

**Tablee 4:** Population-specific F_IS_ indices.

Pop	F_IS_	p value
NW	0.03	0.36
N	0.08	0.18
NE	0.24	0.15
CI	0.18	0
SI	0.08	0.09

### Isolation by Distance

A Mantel test revealed a positive correlation between genetic (DPS, based on proportion of shared alleles) and geographic distances (Mantel’s R = 0.62, p-value = 0.001). This positive relationship was observed only at short distances (Fig. S6). The Mantel’s correlogram (Fig. S7) confirmed this positive relationship over distances of less than 500 km (Mantel’s R = 0.79, p-value = 0.001). Further increase in geographic distance did not result in increase in the genetic distance.

### Genetic Diversity

**Figure 4:**
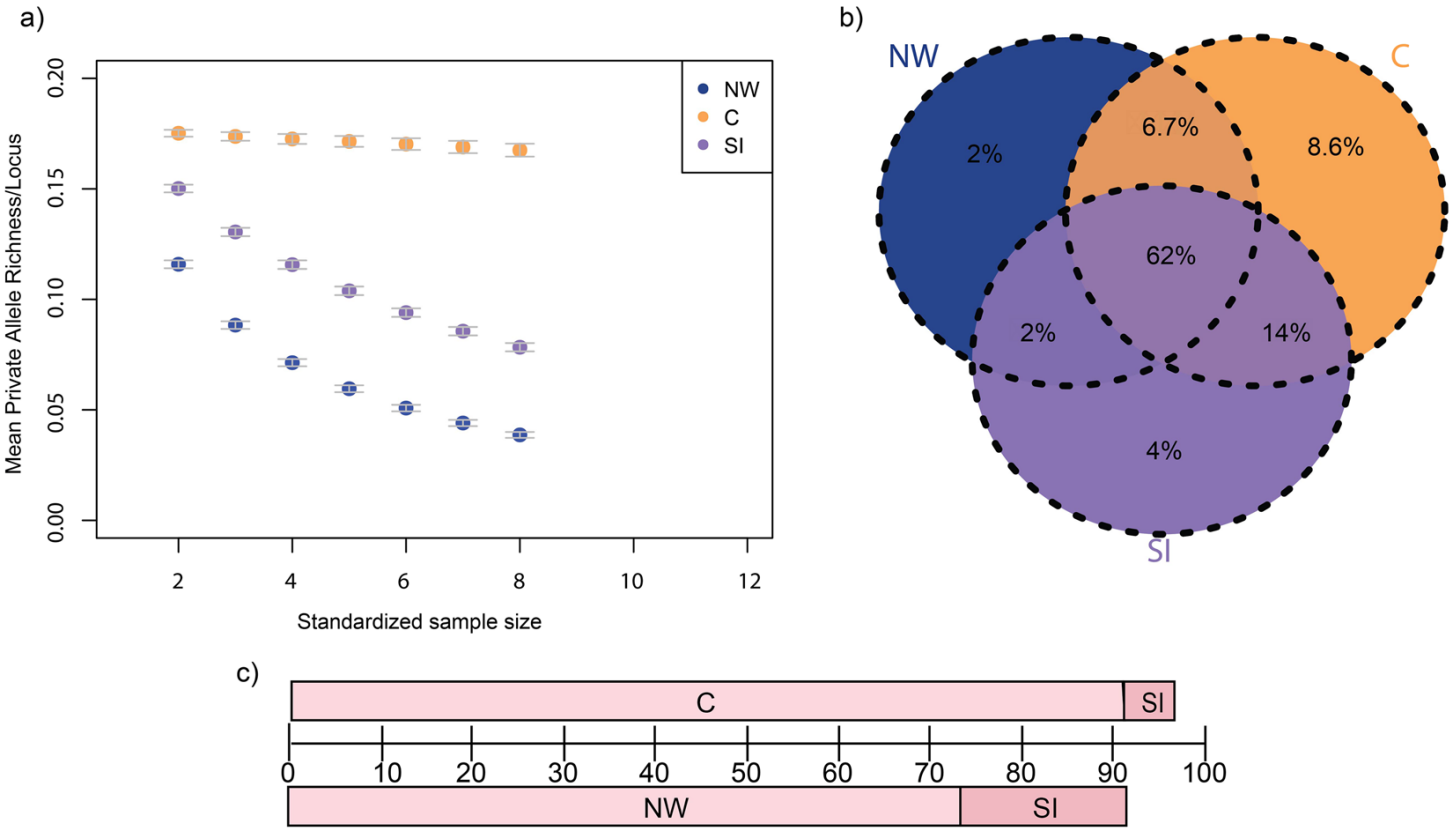
Private and shared genetic diversity between the genetic clusters. (**a**) Mean private allelic richness of each cluster (Y axis) at different sample sizes of alleles (X axis). Alleles were sub-sampled for all possible combinations of loci. The error bars represent standard error values. (**b**) A Venn diagram depicting proportion of private and shared richness in each cluster and their combinations. (**c**) Using the data from the Venn diagram, a bar plot depicting the proportion of genetic diversity retained for two different combinations of clusters.

For almost all sample sizes, NW had the lowest genetic diversity and C had the highest (Fig. 4a). Much of the diversity (Venn diagram, Fig. 4b) was shared across all groups (~62%) with private variation being much lower (NW - 2%, SI - 4% and C - 8.6%, when considering three clusters). Individually, the NW and C clusters represented the lowest and the highest proportion (~73% and ~91% respectively) of total diversity in Indian tigers. Therefore, the combined diversity of NW and SI accounts for about 91% of the total genetic diversity (Fig. 4c). However, CI and SI together account for 97% of the total diversity.

### Climate and vegetation based differentiation of tiger reserves

The PCA including all points across India showed a gradient of separation of tiger reserves along PC2 – mainly driven by precipitation (Figs S13 and S14). When only points falling within tiger reserves were plotted, the protected areas showed a north to south gradient, while the northeast regions showed separation along a different axis (Fig. S16). Multiple temperature and precipitation variables contributed to the two PC axes (Fig. S15).

### Loci under Selection

The Bayescan analysis did not identify any outlier loci (Fig. S8) at a false discovery rate (FDR) of 0.05.

## Discussion

In this study, we investigated population differentiation and genetic diversity of Indian tigers using 10,184 SNPs typed for 38 wild individuals from 17 protected areas (PAs). Bayesian clustering, PCA plots and isolation by distance tests establish genetic differentiation within the Indian subcontinent. Our data and analyses reveal that tiger populations in India cluster into three genetic groups that broadly map onto geographic tiger landscapes and represent groups of proximate protected areas. A large proportion of genetic diversity is shared between clusters, while population specific diversity is variable but low. Importantly, from a conservation perspective, our study reveals an isolated tiger population with low genetic diversity.

Our analyses reveal that tigers from the NW cluster (Ranthambore) are genetically isolated. Such genetic isolation is supported by the fact that they form a distinct cluster in structure analysis (Fig. 2a) and have the highest pair-wise F_ST_s (Table 2) with other genetic clusters. They separate from other individuals in the network analysis, even at low thresholds of genetic similarity (Fig. 2, 0.16). Geographically, Ranthambore is poorly connected to other tiger conservation landscapes^30^. Historically, tigers extended further northwest of Ramthambore into Pakistan, but went locally extinct in the early 1900s^31^. Genetic data reveal that northwest tigers and these extinct populations were connected to other tiger populations in the past^20^. More recently, tiger populations from the two closest PAs to Ranthambore, Panna and Sariska, were extirpated (2009 and 2004 respectively)^32, 33^. Together, this makes Ranthambore the western-most extant population of wild tigers today.

Our data also suggest that genetic diversity in Ranthambore is much lower in comparison to all other genetic clusters (theta (H) = 0.21), possibly due to small effective size and/or isolation. Low genetic variation contributes to higher extinction risk in wild populations^14^. Deleterious mutations also accumulate in small populations (e.g. Woolly mammoths (*Mammuthus primigenius*)^34^). While we point out that our data contain some related individuals (as measured by the p^ statistic, Fig. S4), we reanalyzed population structure excluding these (supplementary materials). Ranthambore individuals (2 samples) still form a separate cluster at the most likely value of K (Fig. S18). Our results suggest that the Ranthambore population is isolated and that inbreeding depression is a realistic possibility in this population. Future research efforts can further investigate this possibility.

Samples from southern India form a distinct genetic cluster (supported by Admixture, PCA, network analysis, and high pair-wise F_ST_). This is in contrast to a previous microsatellite-based study^20^ that found no evidence for differentiation within peninsular India, but consistent with a report on tiger monitoring in India^25^. Our genome-wide SNP data was able to reveal this cryptic population structure^35, 36^. We caution that our final dataset did not include the only large PA in the Eastern Ghats (Nagarjunsagar Srisailam Tiger Reserve - NSTR, Fig. 1), a population that could mediate connectivity between South and central Indian clusters. Admixture analysis with one sample from NSTR was not conclusive as very few reads from the sample passed quality filtering (Fig. S12).

Northeastern tigers were previously assigned to a common cluster with central India^23^. In contrast, a recent report that extensively sampled northeast tigers suggests that they form a distinct genetic cluster^25^. Owing to our limited sampling in this region, our data is unable to resolve the genetic status of northeastern tigers. These individuals show varied genetic affinity in different analyses – part of the central cluster (Admixture analysis), an independent cluster (phylogenetic network) and part of a North – northeast cluster (the network analysis). Further, tigers from this region also have high genetic diversity (high heterozygosity – Table S5 and F_IS_– Table 4). Northeast tigers could be genetically closer to southeast Asian tigers. Evaluating range-wide genomic diversity for tigers (including samples from southeast Asia) may better resolve the population status of the northeast tigers.

The central genetic cluster harbors the highest diversity. High effective size and/or gene flow with neighbouring clusters could explain this. Our results are also consistent with theoretical^37^ and empirical studies (based on microsatellite data) that show greater diversity in centrally located populations, including in endangered species, such as the Cross River gorilla^38^ (*Gorilla gorilla diehli*) and violets^39^ (*Viola pumila*, *V*. *stagnina*).

Despite relatively continuous historical tiger distributions, we observe a signal of genetic isolation by distance. Ecological theory predicts that large-bodied carnivores like tigers may move far (~450 km^40^). On the other hand, a genetic study found support for even higher dispersal distances (~650 km^41^). Our results support ecological theory, with isolation by distance operating at the scale of 300–400 km (Fig. S6). However, loss of connectivity and genetic differentiation can be observed at short distances, as in the case of Ranthambore, and even in central India, as has been reported in previous studies^42, 43^. Overall, if functional connectivity (e.g. through corridors) is maintained between PAs within the identified (or between) clusters, further genetic differentiation can be avoided.

The widely distributed North American grey wolf (*Canis lupus*) occurs over varied environments and reveals signatures of local adaption despite high gene flow^44^. Tigers occur across a range of vegetation types and climatic gradients across India. However, while we do find high genetic differentiation among the clusters, we do not find evidence of adaptive divergence (based on outlier tests). In summary, the clusters identified do not appear to merit the status of conservation units^45^. Our analyses suggest that assisted gene flow between genetic clusters could potentially be a viable conservation strategy if genetic rescue is required. We caution that methods such as ddRAD usually sample only about 1–10% of the genome^46^ and data from whole genomes may be better able to identify signatures of selection.

Population structure can arise through the interplay of multiple factors including population history, selection and connectivity. Disentangling the effects of each of these is often challenging. However, understanding their relative contributions can have important consequences for species survival. For instance, while it is generally understood that small populations are at greater risk of inbreeding and extinction, the duration of time for which the population has been small may have important consequences. Recently bottlenecked populations may be of greater conservation concern than populations that have remained small for a long time^47^. Further, understanding whether population change has occurred in response to older climatic fluctuations, as opposed to more recent anthropogenic effects is important to consider when deciding endangerment status (IUCN criteria) and planning species recovery^45^.

In the case of Indian tigers, we find evidence for population structure. While the southern Indian cluster includes several populations, the northwestern cluster is restricted to a single tiger population, Ranathambore. The observed genetic differentiation between Ranathambore and the central cluster could signify the lack of gene flow relatively recently and/or older population divergence. Additionally, differential population size changes through historical time could impact the magnitude of differentiation. We attempted to understand the demographic history of the genetic clusters we identified using a site frequency spectrum-based approach. However, the estimated site frequency spectrum had very few singletons and none of the models fit our data well (supplementary materials, Fig. S19). This could be due to a combination of our small sample size, as well as the fact that tigers from the Indian subcontinent have undergone a very recent bottleneck about 200 years ago^23^, making it difficult to accurately infer demographic history^48^. In summary, we cannot explain which parameters (changes in population size, changes in connectivity, or both) are responsible for the northwestern differentiation we observe. Whole genome sequencing and higher sample sizes may allow better detection of low frequency alleles and more accurate reconstruction of demographic history in the future.

Because it is difficult to sample wild tigers invasively, our sampling is opportunistic. As a result, our sample sizes are low and tend to be clustered in space, both of which may impact our results^49, 50^. It is possible that we may be underestimating the number of genetic clusters due to underrepresentation of populations. Similarly, higher sample sizes in the northeast may resolve admixture signatures^51^. However, while our sample sizes are small, the number of loci is high, providing greater power to detect population structure^50, 52^. Future studies that acquire genome-wide data from non-invasive sources such as tiger scat will enhance our ability to sample geographically^53^, allowing us to detect existing and on-going changes in population structure and connectivity.

Population genomics of wild species is being increasingly used to inform conservation practice, including to make recommendations on reintroduction (e.g. wolves (*Canis lupus*)^54^), to set up breeding programs (e.g. Tasmanian devils (*Sarchophilus harrisii*)^7^) and to designate management/conservation units^55^. Our population genomic study of wild Indian tigers finds evidence for a small and isolated population of tigers in northwest India. The long-term persistence of this population may require connectivity with neighboring populations, such as with central India. In summary, our study reveals how genome-wide data and analyses can flag populations that may require urgent conservation attention (such as Ranthambore) and identify strongholds of variation (such as central India). Such inference has significant impacts for on-ground conservation practice.

## Methods

### Samples and DNA Extraction

Tissue samples of wild tigers are difficult to obtain since capturing them is logistically challenging in India. However, multiple laboratories working on tiger population genetics have access to post-mortem samples and these constitute the bulk of our sampling. In some cases, samples were obtained from captured tigers (e.g. individuals involved in conflict). The list of samples is provided in supplementary Table S1. Geographical locations of samples are shown in Fig. 1. We were able to acquire a total of 54 samples, 50 samples representing 17PAs (out of 49 tiger reserves) and four samples from outside PAs. Samples were preserved in absolute ethanol and stored at −20 °C. DNA extraction was conducted using the spin column method following the manufacturer’s instructions (Qiagen).

### Library preparation

Double-digested RAD libraries were prepared as outlined in Peterson *et al*.^56^. A combination of two frequent cutters, Nla III and MluC I (NEB), was used to maximize coverage by getting more fragments. DNA concentration of all extracts was measured using Qubit (Invitrogen) and the initial quantity for library preparation was standardized to 200 ng. DNA quality was not uniformly high, especially in samples that had been preserved for a long time. Some samples were processed despite poor quality if they were single representatives of their population and were usually included in the library twice. Extended ligation was carried out for 13 hours, as standardized by Chattopadhyay *et al*.^51^. Library quality was assessed based on the bioanalyzer profile. A total of ten libraries was prepared and sent for paired-end sequencing on three lanes of an Illumina HiSeq. 1000 machine. The low diversity issue encountered with ddRAD samples during sequencing was circumvented by the addition of 30% phiX genome into the sequencing run (as suggested by Illumina).

### Processing the Raw Data

The sequencing quality of reads was initially assessed using the program FastQC (http://www.bioinformatics.babraham.ac.uk/projects/fastqc/). The data was demultiplexed into individual samples using the program Stacks^28^. Reads with one mismatch in the index were rescued. Demultiplexed data were filtered to remove reads with poor quality (option –q) and the presence of uncalled bases (option –c), and were also truncated to 80 base pairs (option –t). The read numbers retained per sample varied considerably. The quality-filtered reads were aligned to the reference genome^57^ using Bowtie2^58^. Both paired and unpaired reads were aligned with default criteria. Technical replicates were merged and Samtools^59^ was used to process the aligned data. Read group information was added using Picard tools (http://picard.sourceforge.net) to reflect the unique sample identity. Reads were realigned to account for indels using the GATK package^60^. Since data from samples that had been sequenced twice were combined after alignment, the number of unique loci/regions identified in each sample (instead of raw reads per sample) was used to compare data obtained per sample. Depth and coverage stats were calculated using Stacks and Bedtools^61, 62^.

### SNP Calling and Filtering

Unique regions were identified in the program Stacks for each sample. Samples that had fewer than 100,000 loci were eliminated from further analysis. Our data had low average depth (Supplementary Fig. S1), and therefore Freebayes^29^ was used to call SNPs, as it considers multiple samples from a population simultaneously to call variants with high confidence. Only reads supported by at least 8x depth of coverage in the population were considered to identify an allele. Additionally, only dinucleotide variants (excluding indels, MNPs and complex variants) were retained. To filter loci with low data, we calculated the missing data per sample using vcftools^63^. To optimize the tradeoff between the number of samples retained and the number of loci with low missing data, the dataset was filtered multiple times. The vcf file obtained was subjected to filtering based on missing data (<5% per SNP), mapping quality (>= 30) and minor allele frequency (>= 0.05). Thinning of loci within 500 bp of each other was done to remove SNPs that might be in linkage disequilibrium. SNPs that were not in Hardy-Weinberg equilibrium (p < 0.01) were filtered out. Additionally, SNPs were also filtered based on mean depth of coverage across samples. However, as the results obtained with this set of loci (7741 SNPs) were qualitatively similar to those observed with the larger dataset (comparison of datasets based on F_ST_ and Admixture results), the smaller dataset was not utilized in the final analysis (data not shown).

### Genetic Differentiation

Multiple methods were used to infer tiger genetic clusters. A phylogenetic network was constructed in the program SplitsTree4^64^. A NeighborNet network was computed based on p-distance generated from the input data in the form of fasta sequences. A maximum likelihood based approach implemented in the program Admixture^65^ was used to identify the number of population clusters supported by the data. The program was run with 10-fold cross-validation for K values ranging from 1 to 6. The best K was inferred based on the value of K that had the least cross-validation error. For a more visual interpretation of population structure, a principal components analysis (PCA) was performed using an R package^66^. Additionally, a network-based approach was also used to infer hierarchical clustering as implemented in NetStruct^67^. The program constructs a network by connecting individuals above a genetic similarity threshold. This is followed by community detection to identify groups of individuals much more closely related to each other than to those in other groups – a process analogous to detecting genetic clusters. Based on the observed range of genetic distance, the network was plotted at multiple thresholds, followed by community detection using a fast-greedy algorithm. A permutation test was done to test the significance of the observed network.

Pair-wise F_ST_ was calculated between clusters using the Weir and Cockerham estimator in Arlequin^68^. Summary statistics, including heterozygosity (He) values and theta (H), were computed in Arlequin^68^. Pair-wise relatedness (p^) and individual inbreeding coefficients (f) were estimated in Plink^69^. AMOVA^68^ was used to test for hierarchical structure. Theta (H) was estimated as a proxy for effective size (Ne) of each genetic cluster.

### Isolation by Distance

A Mantel test was performed to check for isolation by distance in R^66^. Genetic (DPS = 1− proportion of shared alleles) and geographic distance were calculated for all pairs of individuals. To test for significance of the observed Mantel’s R a randomization was done with 999 replicates. A Mantel’s correlogram (package vegan) was used to identify the distance classes at which the correlation was significant.

### Distribution of Genetic Diversity between Clusters

To see how genetic diversity is distributed across the populations, private allele richness was estimated for each cluster, as well as for combinations of clusters. Since diversity measures can be influenced by sample size, these were standardized across clusters using rarefaction. Private allele richness was estimated for each standardized sample size using the program ADZE^70^ and plotted. To visualize sharing of diversity, the results were plotted as a Venn diagram by calculating the proportion for total diversity in each (or combination of) cluster(s).

### Climate and vegetation based differentiation of tiger reserves

In order to visually inspect whether the available habitats in tiger reserves were differentiated based on climate and/or vegetation, a PCA was run for 19 bioclimatic variables (bioclim), aridity (FAO Global Aridity Index http://www.fao.org/nr/aboutnr/nrl/en/) and land cover classification (GlobCover Land Cover v2.2 - http://geodata.grid.unep.ch/options.php?selectedID = 2054&selectedDatasettype = 16). Regular points were placed on an outline of the India map (grid spacing 0.1) in QGis (v2.0.1). Multiple climate layers (http://www.worldclim.org/bioclim), aridity and vegetation (GlobCover) were used to define the environmental space for the sampled points. The ‘point sampling tool’ was used to extract data for the previously generated points. Points falling within tiger reserves were marked in the attribute table. This table was exported to the software R (v3.3.2) and after centering and scaling the data, a PCA was performed with all points. To closely examine the separation between the tiger reserves, a PCA of only points falling in tiger reserves was performed.

### Test for Loci under Selection

A Bayesian approach was used to test for SNPs that might be under selection among the different genetic clusters identified using the software tool Bayescan^71^. A 5% false discovery rate was used to identify outlier loci. The Bayescan analysis was run with prior odds for the neutral model set to 1000; higher odds lower the possibility of obtaining false positives. It is recommended to set the prior odds high (1000) when using thousands of markers.

### Data Accessibility

The sequences are available on GenBank and can be obtained from the SRA database with the accession number SRP114885.

### Ethical Approval

Our study does not involve any experiments with live animals. Further, while we use tissue samples, none of these were directly obtained for the purpose of this study. We only used samples from tissue previously collected for other studies/purposes. Therefore, ethical clearance regarding sample collection is not applicable to our study.

## Electronic supplementary material

Supplementary Materials: Conservation priorities for endangered Indian tigers through a genomic lens
