# Supplementary material for: Conservation priorities for endangered Indian tigers through a genomic lens
Source: Sci Rep. 2017 Aug 29;7:9614. doi: 10.1038/s41598-017-09748-3 (PMC5575265; doi:10.1038/s41598-017-09748-3)
Supplement: Supplementary file 1 — Supplementary Materials: Conservation priorities for endangered Indian tigers through a genomic lens [file 41598_2017_9748_MOESM1_ESM.pdf]

Supplementary Materials

Conservation priorities for endangered Indian tigers through a genomic lens

Meghana Natesh[1, 2*], Goutham Atla[1], P. Nigam[3], Y. V. Jhala[3], Arun Zachariah[4], Udayan Borthakur[5] and Uma Ramakrishnan[1*]

I) SNP calling and selecting samples for analysis

The total number of samples (tissue/ blood) obtained for the study was 54 (table S1). Depth was initially calculated in the program Stacks[1] from the log file generated at the end of the ref_map.pl script. For each sample, mean coverage depth value was extracted from this file, and has been plotted in figure S1. The number of unique loci identified per sample has been plotted in figure S2. Here, four samples (Corbett -1, Sunderbans, Melghat and NSTR) did not have any reads matching the catalog (in Stacks) and hence were not plotted. For the SNPs called in Stacks, relatively few SNPs passed the minimum depth criteria for the allowed level of missing data (depth=5, missing data allowed=30%). Therefore, we used Freebayes[2] to call SNPs, which uses a multi-sample approach for calling SNPs. A few of the samples discarded based on the number of loci obtained in Stacks (<100,000) could be rescued as a result. The depth was calculated based on these SNPs using the Bedtools[3] genome coverage tool. For each sample, the number of sites represented at increasing depth shows a decreasing trend (figure S3). The final number of samples retained in the analysis was 39 (after filtering criteria based on missing data) and the missing data per sample is shown in table S4. Discarded samples are listed in table S3.

Some of the samples discarded from our dataset were single representatives from their protected areas. We attempted to retain these samples with low data (very few reads mapping to the tiger genome), for a second dataset – retaining more individuals at a few SNPs common across the samples. To do this, we identified regions present in each sample that were in common with SNPs in vcf file from the samples finally used. This vcf file was called allowing for 50% missing data to capture more SNPs. Bedtools was used to identify regions in common with the vcf file for each of these samples (Table S2). The bedfiles were then compared to look for overlap between the samples. However, with the exception of one region that was found to be common between 2 samples, there were no overlaps among the samples with low data.

Since no common regions could be identified, regions identified for each sample were combined into a single bedfile, and these regions were extracted from the larger vcf file containing all the samples. However, this led to a vcf file having over 11,000 SNPs at 20% missing data allowed per SNPs. The missing data for the samples with low data however was extremely high. Hence we were unable to incorporate these samples into the analysis.

Table S1: Number of samples obtained per location. Samples caught outside protected areas are marked by stars.

Sl. No.	Location	Samples	Region
1	Ranthabore	7	North-West
2	Corbett	7	North
4	Arunachal*	1	North-East
5	Kaziranga	2	North-East
6	Morigaon*	1	North-East
7	Simlipal	1	Central
8	Sunderbans	2	Central
9	Panna	2	Central
10	Bandhavgarh	2	Central
11	Nagpur*	1	Central
12	Chandrapur*	1	Central
13	Kanha	7	Central
14	Pench	4	Central
15	Melghat	1	Central
16	Tadoba	1	Central
17	NSTR	1	Central
18	Nagarahole	2	South
19	Bandipur	3	South
20	Wayanad	6	South
21	Satyamangalam	1	South
22	BRT	1	South

Figure S1: Mean coverage depth (Y axis) per sample (X axis) calculated in Stacks for 56 samples. Samples are organized by region for ease of comparison.

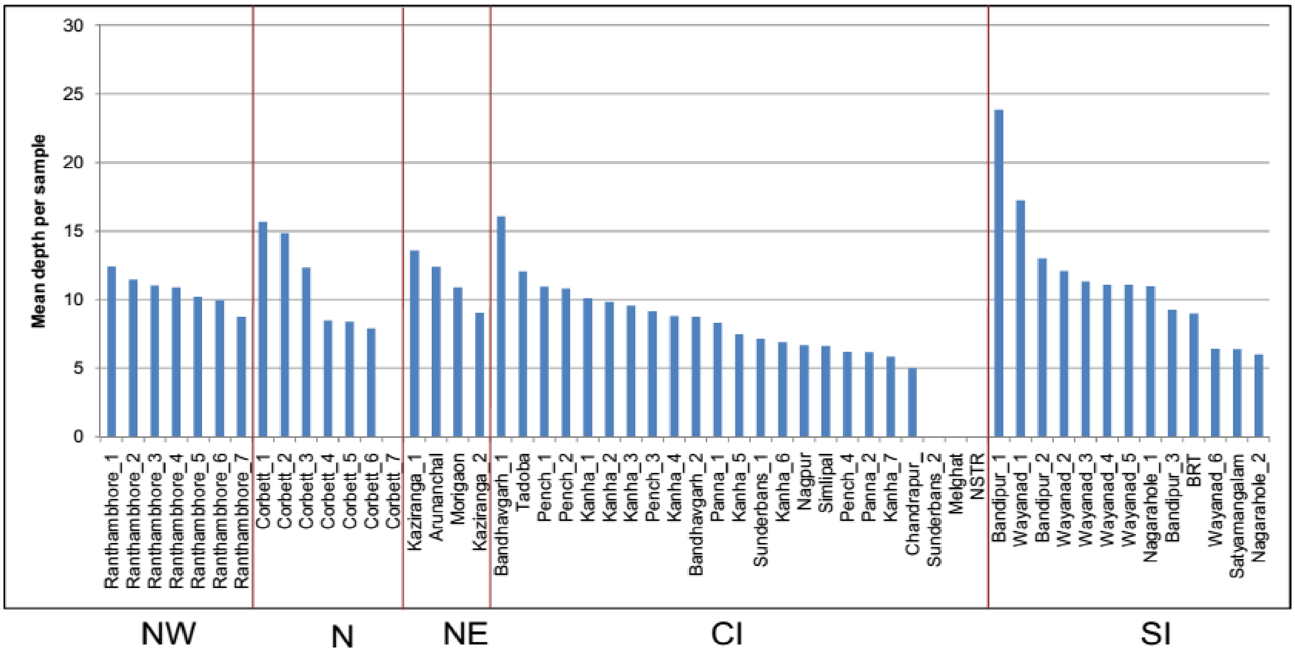

Figure S2: Number of loci per sample as calculated in Stacks. Samples are organized by region for ease of comparison.

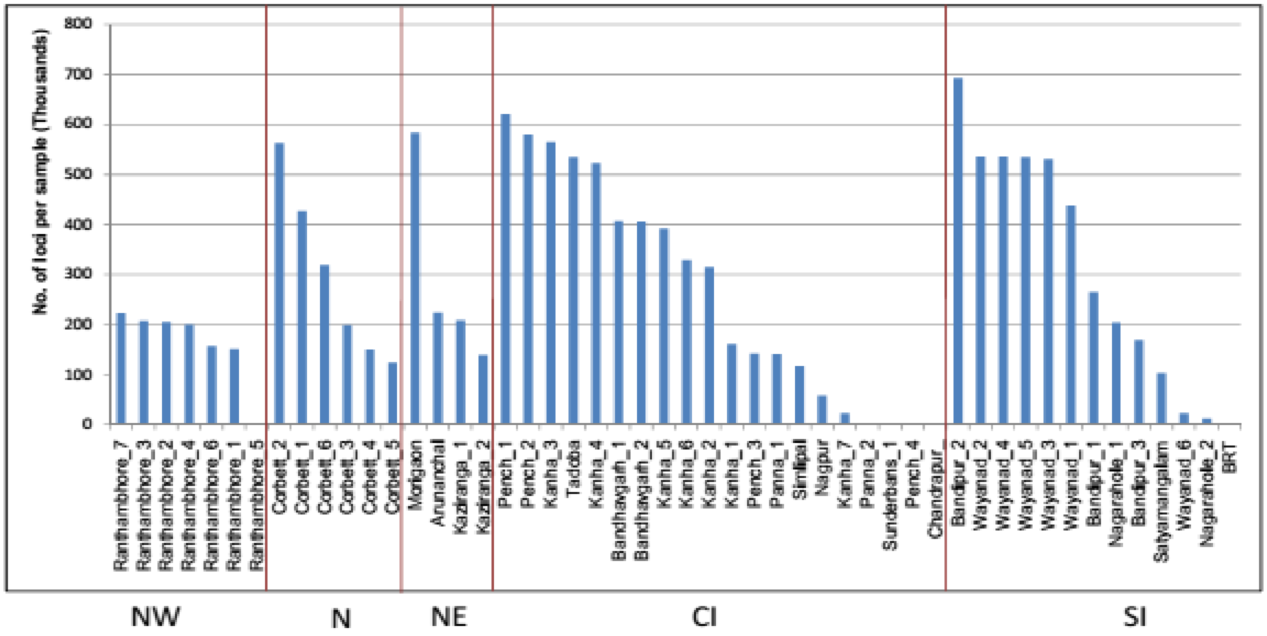

Figure S3: Depth statistics calculated using Bedtools (genome coverage) tool for SNPs identified using Freebayes. The number of regions at a particular depth is depicted on the Y axis in the log scale. For each sample, the number of regions occurring at 4 different values of depth are shown – 3X, 8X, 15X and 20X. Samples are organized by region for ease of comparison.

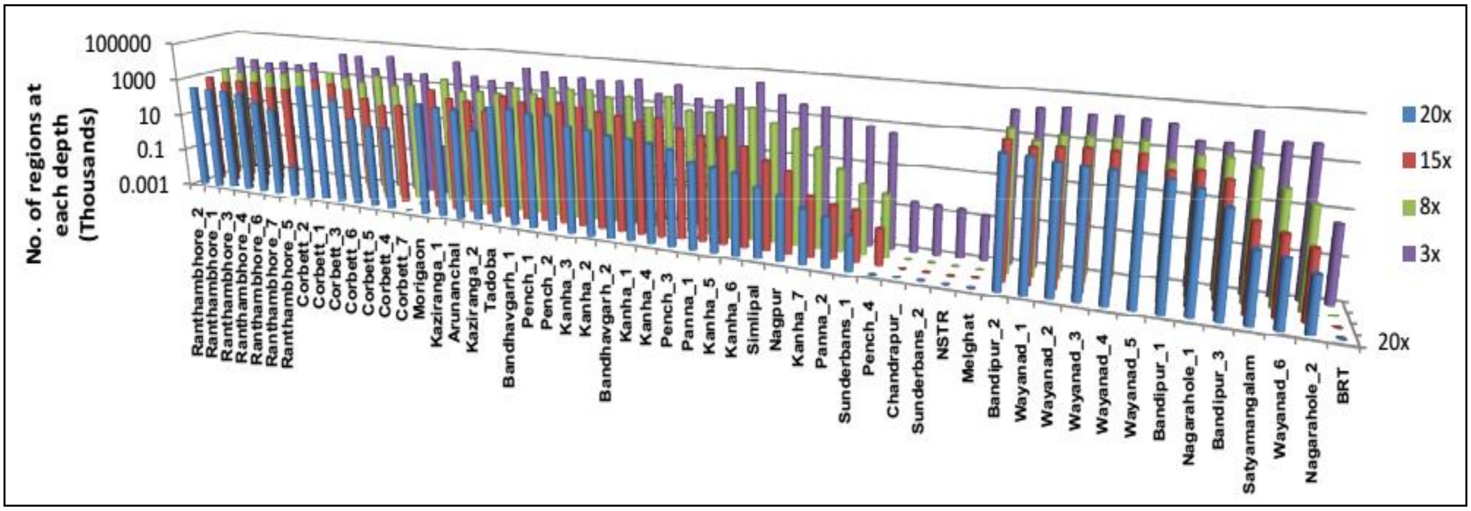

Table S2: Unique regions identified in poor quality samples.

Sample	Total regions	Unique regions
Tadoba	969,044	136,836
Sunderbans	46,438	36,286
BRT	3,360	3,213
Corbett	1,431	1,406
NSTR	987	970
Melghat	513	507
Chandrapur	483	480

Table S3: List of samples excluded from the final analysis.

Sample	Reason for being discarded from analysis
Ranthambhore_2	Recapture
Ranthambhore_5	Low data
Corbett_7	Low data
Sunderbans_1	Low data
Sunderbans_2	Low data
Bandhavgarh_1	Low data
Chandrapur	Low data
Kanha_2	Low data
Melghat	Low data
Nagpur	Aligns with N cluster, even though geographically part of CI. However, protected area of origin unverifiable.
Pench_4	Low data
Tadoba	Low data
NSTR	Low data
BRT	Low data
Wayanad_1	Low data
Wayanad_6	Low data

Table S4: Percentage of missing data per sample in the final list of samples.

Sl No.	Sample	Total SNPs	No. Missing	Missing proportion
1	Ranthambhore_6	10184	626	0.06
2	Ranthambhore_3	10184	17	0.00
3	Ranthambhore_4	10184	28	0.00
4	Ranthambhore_1	10184	585	0.06
5	Ranthambhore_2	10184	12	0.00
6	Corbett_6	10184	41	0.00
7	Corbett_2	10184	3	0.00
8	Corbett_1	10184	3	0.00
9	Corbett_3	10184	8	0.00
10	Corbett_4	10184	77	0.01
11	Corbett_5	10184	893	0.09
12	Morigaon	10184	6	0.00

13	Arunanchal	10184	19	0.00
14	Kaziranga_2	10184	752	0.07
15	Kaziranga_1	10184	23	0.00
16	Simlipal	10184	403	0.04
17	Panna_2	10184	2307	0.23
18	Panna_1	10184	52	0.01
19	Bandhavgarh_2	10184	86	0.01
20	Pench_2	10184	14	0.00
21	Pench_1	10184	353	0.03
22	Pench_3	10184	747	0.07
23	Kanha_7	10184	1933	0.19
24	Kanha_6	10184	1306	0.13
25	Kanha_4	10184	479	0.05
26	Kanha_5	10184	1172	0.12
27	Kanha_3	10184	38	0.00
28	Kanha_1	10184	592	0.06
29	Nagarahole_2	10184	1627	0.16
30	Nagarahole_1	10184	35	0.00
31	Bandipur_3	10184	41	0.00
32	Bandipur_1	10184	6	0.00
33	Bandipur_2	10184	6	0.00
34	Wayanad_3	10184	7	0.00
35	Wayanad_2	10184	190	0.02
36	Wayanad_5	10184	7	0.00
37	Wayanad_4	10184	8	0.00
38	Satyamangalam	10184	1513	0.15

II) Estimation of summary statistics (pair-wise relatedness, inbreeding and He) in each population

Relatedness and inbreeding were estimated using the software PLINK[4]. The p^ estimator from PLINK indicated that the samples included a recapture (p^ ~ 0.9). Therefore, one individual from this pair (Ranthambore_2) was removed from all analyses. Pairwise relatedness is plotted as a heat map in figure S4. As can be seen, pairs in Ranthambore have higher relatedness. Inbreeding coefficients for individuals were grouped into 5 regions and have been plotted as a boxplot in figure S5. While the inbreeding coefficient is highly variable within each group, Ranthambore (NW) shows overall high values. Expected heterozygosity for a five cluster scenario is presented in Table S6.

Figure S4: Pairwise relatedness (p^) as estimated in PLINK between all pairs of individuals within a genetic cluster.

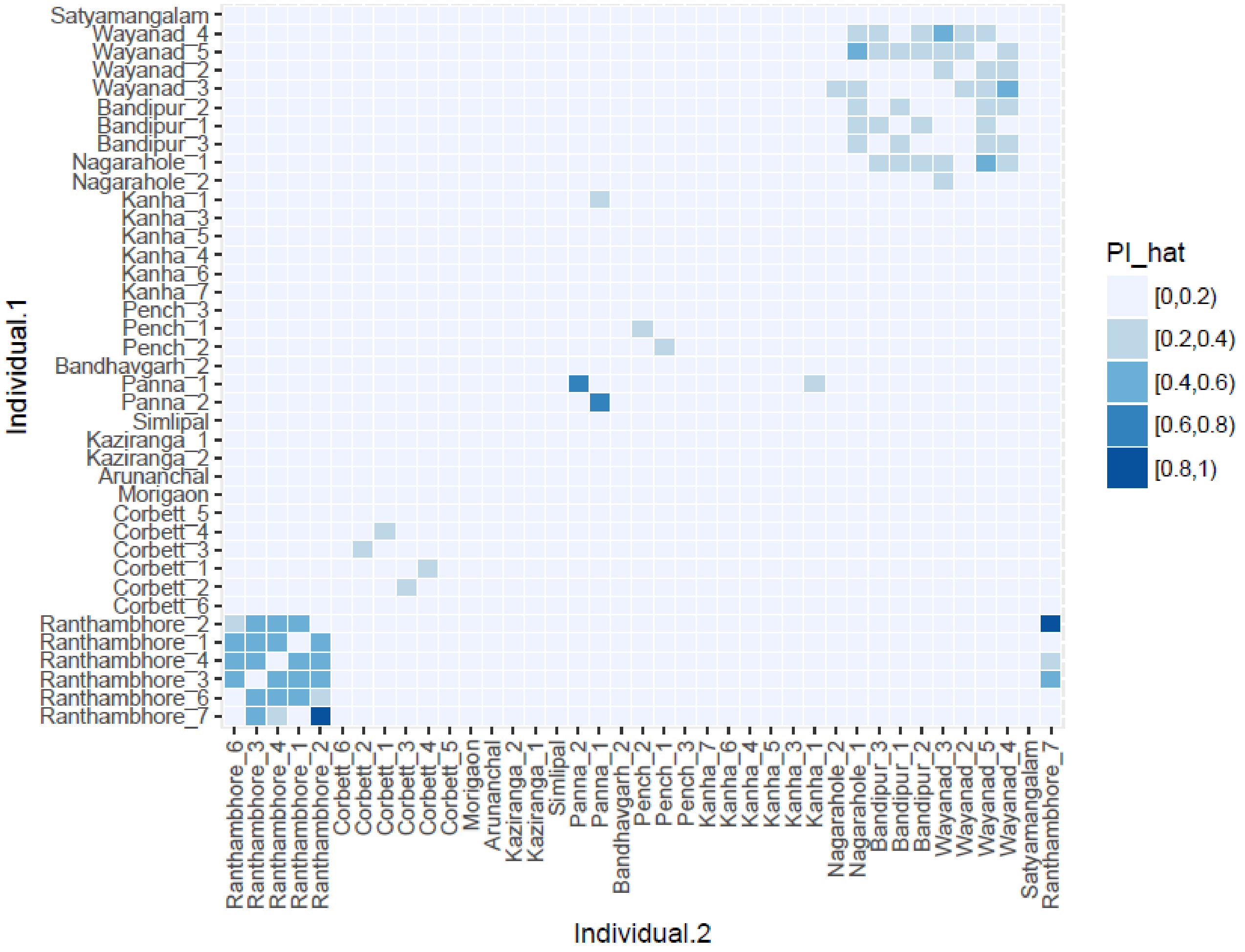

Figure S5: Inbreeding coefficients estimated in Plink.

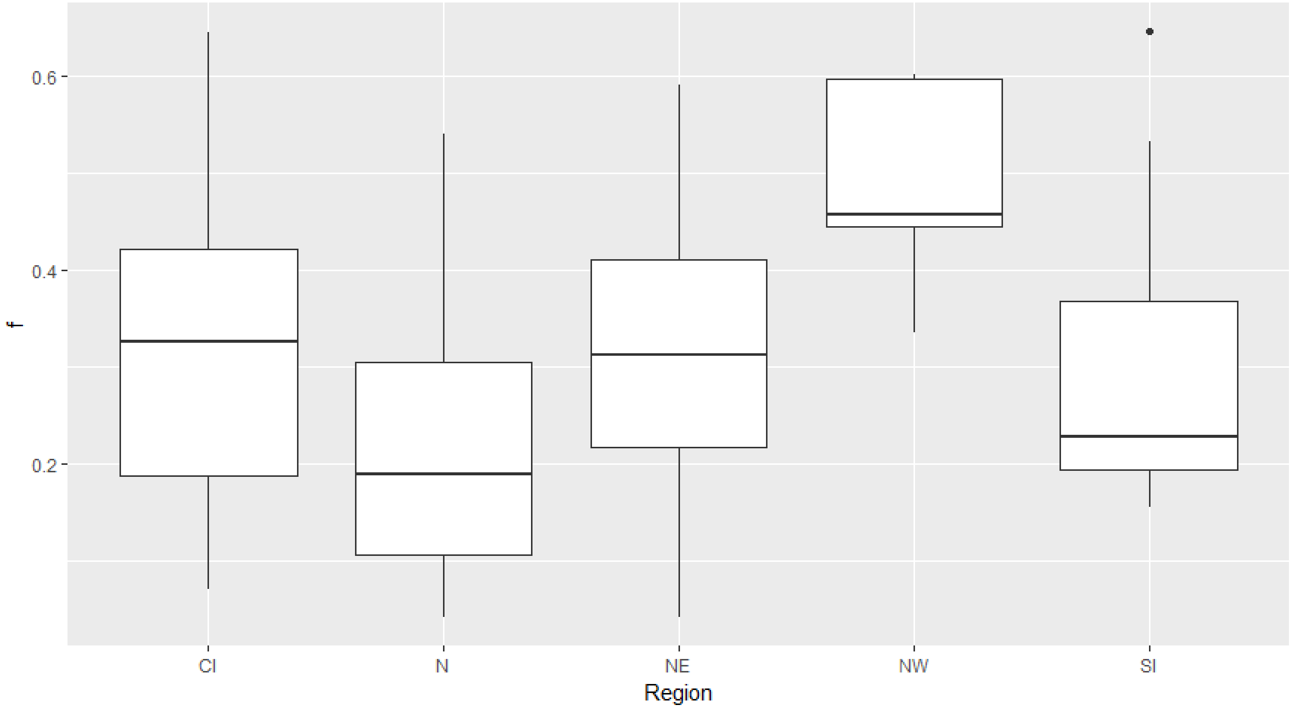

Table S5: Expected Heterozygosity (He) considering five clusters, estimated using Arlequin[5].

	NW	N	NE	CI	SI
Mean	0.17894	0.28111	0.288	0.29857	0.26051
s.d.	0.21846	0.20933	0.24908	0.19172	0.20363

III) Coefficient of ancestry at different values of K

Table S6: Coefficient of ancestry at K = 2

Ranthambhore_6	0.00001	0.99999
Ranthambhore_3	0.00001	0.99999
Ranthambhore_4	0.00001	0.99999
Ranthambhore_1	0.00001	0.99999
Ranthambhore_2	0.00001	0.99999
Corbett_6	0.039174	0.960826
Corbett_2	0.061004	0.938996
Corbett_1	0.064301	0.935699
Corbett_3	0.045047	0.954953
Corbett_4	0.055198	0.944802
Corbett_5	0.00001	0.99999
Morigaon	0.139436	0.860564
Arunanchal	0.192786	0.807214
Kaziranga_2	0.134791	0.865209
Kaziranga_1	0.118905	0.881095
Simlipal	0.173627	0.826373
Panna_2	0.00001	0.99999
Panna_1	0.00001	0.99999
Bandhavgarh_2	0.138653	0.861347
Pench_2	0.089483	0.910517
Pench_1	0.088185	0.911815
Pench_3	0.107326	0.892674
Kanha_7	0.042865	0.957135
Kanha_6	0.050798	0.949202
Kanha_4	0.029667	0.970333
Kanha_5	0.022518	0.977482
Kanha_3	0.049858	0.950142
Kanha_1	0.00001	0.99999
Nagarahole_2	0.99999	0.00001
Nagarahole_1	0.99999	0.00001
Bandipur_3	0.99999	0.00001
Bandipur_1	0.902019	0.097981
Bandipur_2	0.99999	0.00001
Wayanad_3	0.99999	0.00001
Wayanad_2	0.99999	0.00001
Wayanad_5	0.99999	0.00001
Wayanad_4	0.99999	0.00001
Satyamangalam	0.99999	0.00001

Table S7: Coefficient of ancestry at K = 3

Ranthambhore_6	0.00001	0.00001	0.99998
Ranthambhore_3	0.00001	0.00001	0.99998
Ranthambhore_4	0.00001	0.00001	0.99998
Ranthambhore_1	0.00001	0.00001	0.99998
Ranthambhore_2	0.00001	0.00001	0.99998
Corbett_6	0.99998	0.00001	0.00001
Corbett_2	0.961926	0.002567	0.035508
Corbett_1	0.99998	0.00001	0.00001
Corbett_3	0.960335	0.00001	0.039655
Corbett_4	0.995406	0.00001	0.004584
Corbett_5	0.99998	0.00001	0.00001
Morigaon	0.850638	0.090867	0.058495
Arunanchal	0.783994	0.156029	0.059977
Kaziranga_2	0.876087	0.08793	0.035983
Kaziranga_1	0.875149	0.072441	0.052411
Simlipal	0.804178	0.134402	0.06142
Panna_2	0.99998	0.00001	0.00001
Panna_1	0.99998	0.00001	0.00001
Bandhavgarh_2	0.814204	0.0976	0.088196
Pench_2	0.979236	0.016405	0.004359
Pench_1	0.961921	0.024141	0.013938
Pench_3	0.871358	0.059689	0.068953
Kanha_7	0.99998	0.00001	0.00001
Kanha_6	0.99998	0.00001	0.00001
Kanha_4	0.99998	0.00001	0.00001
Kanha_5	0.99998	0.00001	0.00001
Kanha_3	0.992514	0.00001	0.007476
Kanha_1	0.99998	0.00001	0.00001
Nagarahole_2	0.00001	0.99998	0.00001
Nagarahole_1	0.00001	0.99998	0.00001
Bandipur_3	0.00001	0.99998	0.00001
Bandipur_1	0.082948	0.899047	0.018005
Bandipur_2	0.00001	0.99998	0.00001
Wayanad_3	0.00001	0.99998	0.00001
Wayanad_2	0.00001	0.99998	0.00001
Wayanad_5	0.00001	0.99998	0.00001
Wayanad_4	0.00001	0.99998	0.00001
Satyamangalam	0.00001	0.99998	0.00001

Table S8: Coefficient of ancestry at K = 4

Ranthambhore_6	0.99997	0.00001	0.00001	0.00001
Ranthambhore_3	0.99997	0.00001	0.00001	0.00001
Ranthambhore_4	0.999962	0.000018	0.00001	0.00001
Ranthambhore_1	0.999964	0.00001	0.000016	0.00001
Ranthambhore_2	0.99997	0.00001	0.00001	0.00001
Corbett_6	0.00001	0.00001	0.00001	0.99997
Corbett_2	0.00001	0.00001	0.00001	0.99997
Corbett_1	0.00001	0.00001	0.00001	0.99997
Corbett_3	0.00001	0.00001	0.00001	0.99997
Corbett_4	0.00001	0.00001	0.00001	0.99997
Corbett_5	0.00001	0.00001	0.00001	0.99997
Morigaon	0.049838	0.075342	0.314719	0.560101
Arunanchal	0.054956	0.154897	0.372267	0.41788
Kaziranga_2	0.020257	0.069003	0.28499	0.62575
Kaziranga_1	0.040585	0.043942	0.291941	0.623532
Simlipal	0.059266	0.137815	0.485069	0.31785
Panna_2	0.00001	0.00001	0.99997	0.00001
Panna_1	0.00001	0.00001	0.99997	0.00001
Bandhavgarh_2	0.085097	0.09652	0.54118	0.277203
Pench_2	0.00001	0.00001	0.934594	0.065386
Pench_1	0.00001	0.00001	0.940328	0.059652
Pench_3	0.071543	0.051919	0.735329	0.14121
Kanha_7	0.00001	0.00001	0.99997	0.00001
Kanha_6	0.00001	0.00001	0.99997	0.00001
Kanha_4	0.00001	0.00001	0.99997	0.00001
Kanha_5	0.00001	0.00001	0.99997	0.00001
Kanha_3	0.00001	0.00001	0.99997	0.00001
Kanha_1	0.00001	0.00001	0.99997	0.00001
Nagarahole_2	0.00001	0.99997	0.00001	0.00001
Nagarahole_1	0.00001	0.99997	0.00001	0.00001
Bandipur_3	0.00001	0.99997	0.00001	0.00001
Bandipur_1	0.015383	0.902782	0.081825	0.00001
Bandipur_2	0.00001	0.99997	0.00001	0.00001
Wayanad_3	0.00001	0.99997	0.00001	0.00001
Wayanad_2	0.000016	0.999964	0.00001	0.00001
Wayanad_5	0.00001	0.99997	0.00001	0.00001
Wayanad_4	0.00001	0.99997	0.00001	0.00001
Satyamangalam	0.00001	0.99997	0.00001	0.00001

Table S9: Coefficient of ancestry at K = 5

Ranthambhore_6	0.99996	0.00001	0.00001	0.00001	0.00001
Ranthambhore_3	0.99996	0.00001	0.00001	0.00001	0.00001
Ranthambhore_4	0.99996	0.00001	0.00001	0.00001	0.00001
Ranthambhore_1	0.999948	0.000017	0.000015	0.00001	0.00001
Ranthambhore_2	0.99996	0.00001	0.00001	0.00001	0.00001
Corbett_6	0.00001	0.00001	0.99996	0.00001	0.00001
Corbett_2	0.00001	0.00001	0.99996	0.00001	0.00001
Corbett_1	0.00001	0.00001	0.999955	0.000015	0.00001
Corbett_3	0.00001	0.00001	0.99996	0.00001	0.00001
Corbett_4	0.00001	0.00001	0.99996	0.00001	0.00001
Corbett_5	0.00001	0.00001	0.99996	0.00001	0.00001
Morigaon	0.00001	0.00001	0.000011	0.999959	0.00001
Arunanchal	0.029234	0.262381	0.185165	0.39668	0.12654
Kaziranga_2	0.00001	0.00001	0.00001	0.99996	0.00001
Kaziranga_1	0.00001	0.00001	0.00001	0.99996	0.00001
Simlipal	0.050883	0.416703	0.162971	0.243008	0.126435
Panna_2	0.00001	0.99996	0.00001	0.00001	0.00001
Panna_1	0.00001	0.99996	0.00001	0.00001	0.00001
Bandhavgarh_2	0.081228	0.498173	0.172986	0.158223	0.089389
Pench_2	0.00001	0.892697	0.035047	0.072126	0.00012
Pench_1	0.000239	0.895307	0.022137	0.082035	0.000281
Pench_3	0.068899	0.685248	0.072361	0.126223	0.047269
Kanha_7	0.00001	0.99996	0.00001	0.00001	0.00001
Kanha_6	0.00001	0.99996	0.00001	0.00001	0.00001
Kanha_4	0.00001	0.99996	0.00001	0.00001	0.00001
Kanha_5	0.00001	0.99996	0.00001	0.00001	0.00001
Kanha_3	0.00001	0.99996	0.00001	0.00001	0.00001
Kanha_1	0.00001	0.99996	0.00001	0.00001	0.00001
Nagarahole_2	0.00001	0.00001	0.00001	0.00001	0.99996
Nagarahole_1	0.00001	0.00001	0.00001	0.00001	0.99996
Bandipur_3	0.00001	0.00001	0.00001	0.00001	0.99996
Bandipur_1	0.016166	0.079941	0.00001	0.00001	0.903873
Bandipur_2	0.00001	0.00001	0.00001	0.00001	0.99996
Wayanad_3	0.00001	0.00001	0.00001	0.00001	0.99996
Wayanad_2	0.00001	0.00001	0.00001	0.00001	0.99996
Wayanad_5	0.00001	0.00001	0.00001	0.00001	0.99996
Wayanad_4	0.00001	0.00001	0.00001	0.00001	0.99996
Satyamangalam	0.00001	0.00001	0.00001	0.00001	0.99996

IV) Isolation by distance

The presence of Isolation by distance was tested using a Mantel test in Adegenet[6]. As the relationship did not appear to be linear at all distance classes, a Mantel's correlogram was performed.

Figure S6: Correlation of genetic distance (DPS) with geographic distance at different size classes of geographic distance. On the X axis, size classes of geographic distance are shown; Mantel correlation is given on the Y axis.

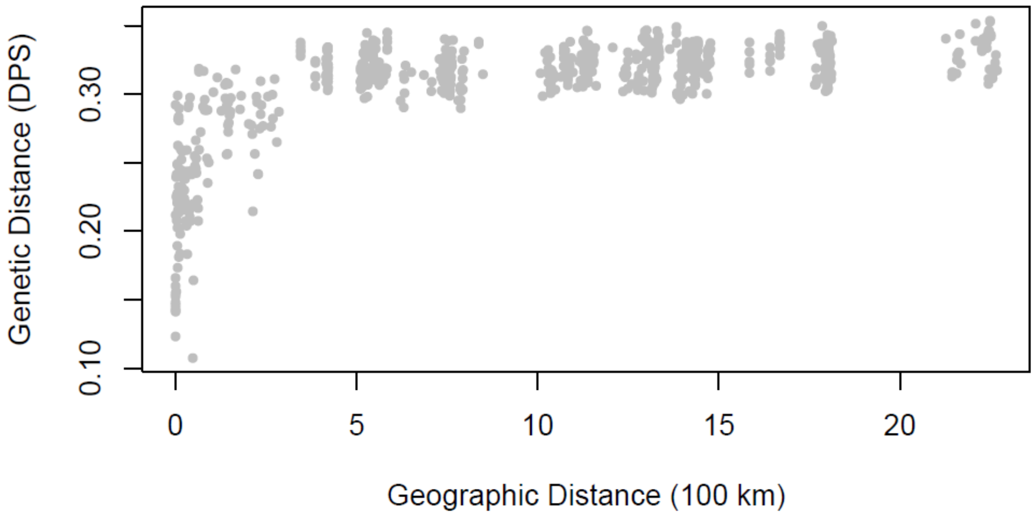

Figure S7: Mantel's correlogram between genetic and geographic distances at different distance classes. On the Y axis is the Mantel correlation and on the X axis is the geographic distance in 1000 km. The black squares indicate significant values (p-value<0.05).

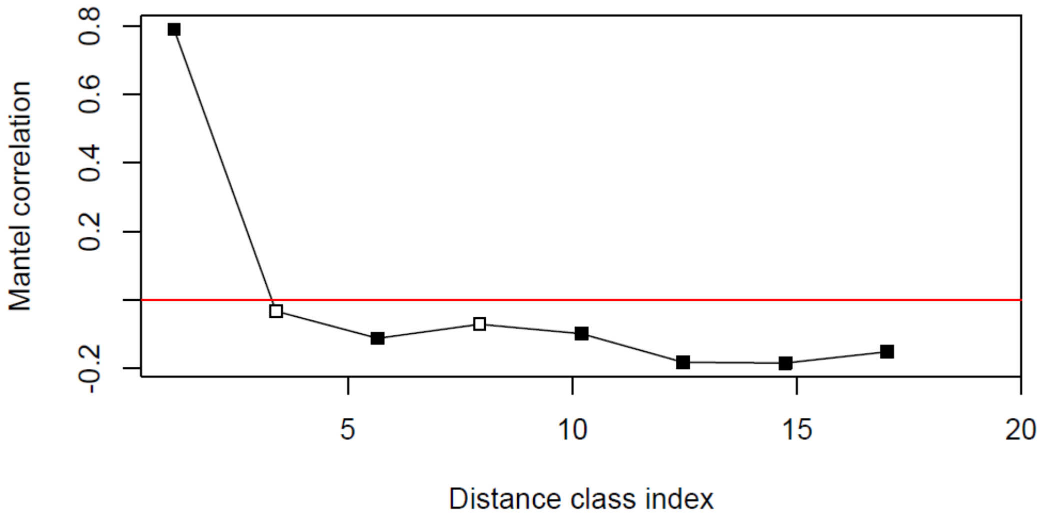

Table S10: Data used to plot Mantel Correlogram, as obtained from the package Vegan in R.

Distance Class	Class Index	N. Dist	Mantel correlation	Pr (Mantel)	Pr (corrected)	Significance
D.cl.1	1.134382	256	0.790323	0.001	0.001	***
D.cl.2	3.40233	108	-0.03302	0.232	0.232	
D.cl.3	5.670279	152	-0.11239	0.009	0.018	*
D.cl.4	7.938227	124	-0.07134	0.047	0.094	.
D.cl.5	10.20618	126	-0.09889	0.011	0.036	*
D.cl.6	12.47412	204	-0.18253	0.001	0.006	**
D.cl.7	14.74207	206	-0.18457	0.001	0.007	**
D.cl.8	17.01002	150	-0.15179	0.001	0.008	**
D.cl.9	19.27797	0	NA	NA	NA	
D.cl.10	21.54592	80	-0.15717	0.001	NA	

V) Test for loci under selection/ outlier loci

Figure S8: Loci potentially under selection identified using an outlier test (Bayescan[7]). The X axis depicts the posterior odds, and the Y axis is F_{ST}. The vertical bar separates outliers (Log10(PO) > 0 from the rest of the loci.

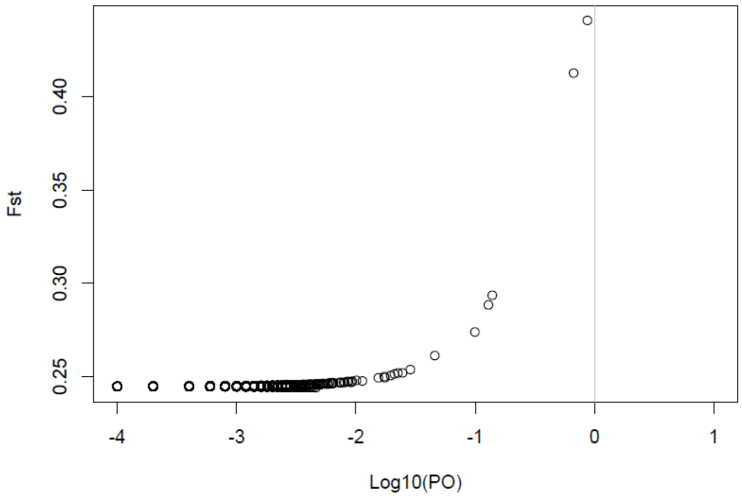

VI) Private allele richness analysis repeated assuming five clusters

When considering five clusters, resampling could only be done up to 6 times as the sample size from NE was a limiting factor. While estimates of private allele richness may not have reached an asymptote for all populations, NW consistently has lower richness. Central India was also tested separately to test if the variation had been completely sampled (Figure S10).

Figure S9: Private allele richness estimated at increasing sample sizes for each population

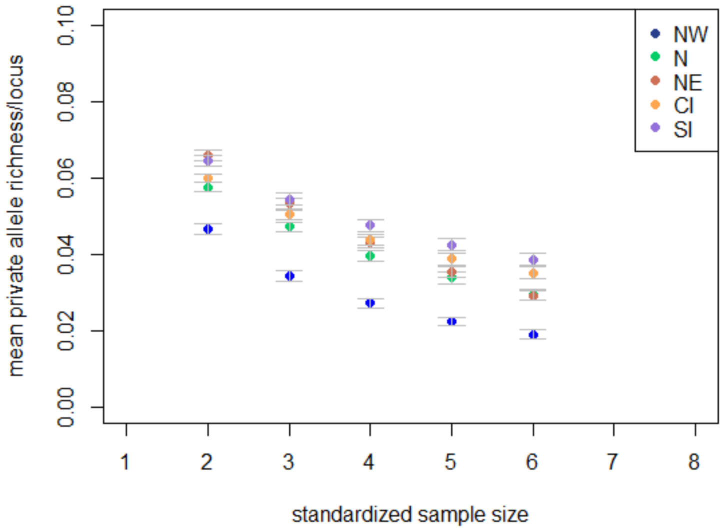

Figure S10: Private allele richness estimated at increasing sample sizes for central India alone.

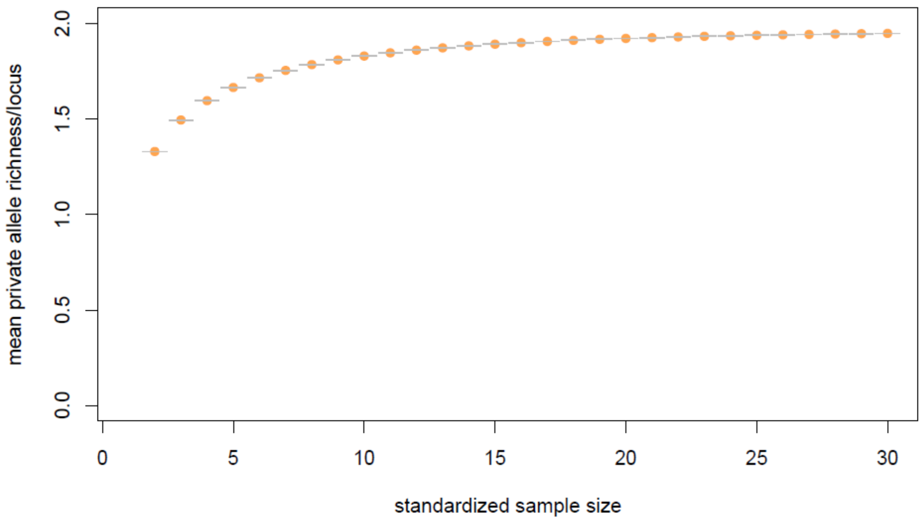

VII) Distribution of strength of association in the network

Community detection in network analysis (NetStruct[8]) assigns each individual to a community. However, individuals may differ in their affinities to the assigned community, reflecting similarity to other clusters. In figure 3a (i), two central Indian tigers are assigned to the NW cluster. However, it can be seen in figure S11 (i) that the strength of association of these individuals to the NW cluster is very low. Similarly, the N and CI clusters at higher thresholds have individuals which have low affinity to the assigned cluster. This likely reflects hierarchical structure, as at lower thresholds, N, NE and CI are assigned to a single C cluster.

Figure S11: Strength of Association Distribution for each inferred cluster (NetStruct). Cluster numbers are shown on the X axis and association to the cluster on the Y axis. Colours have been matched to figure 3 for clarity. The genetic similarity thresholds at which community detection was performed in each instance are depicted above each panel and correspond to the networks in Figure 3a.

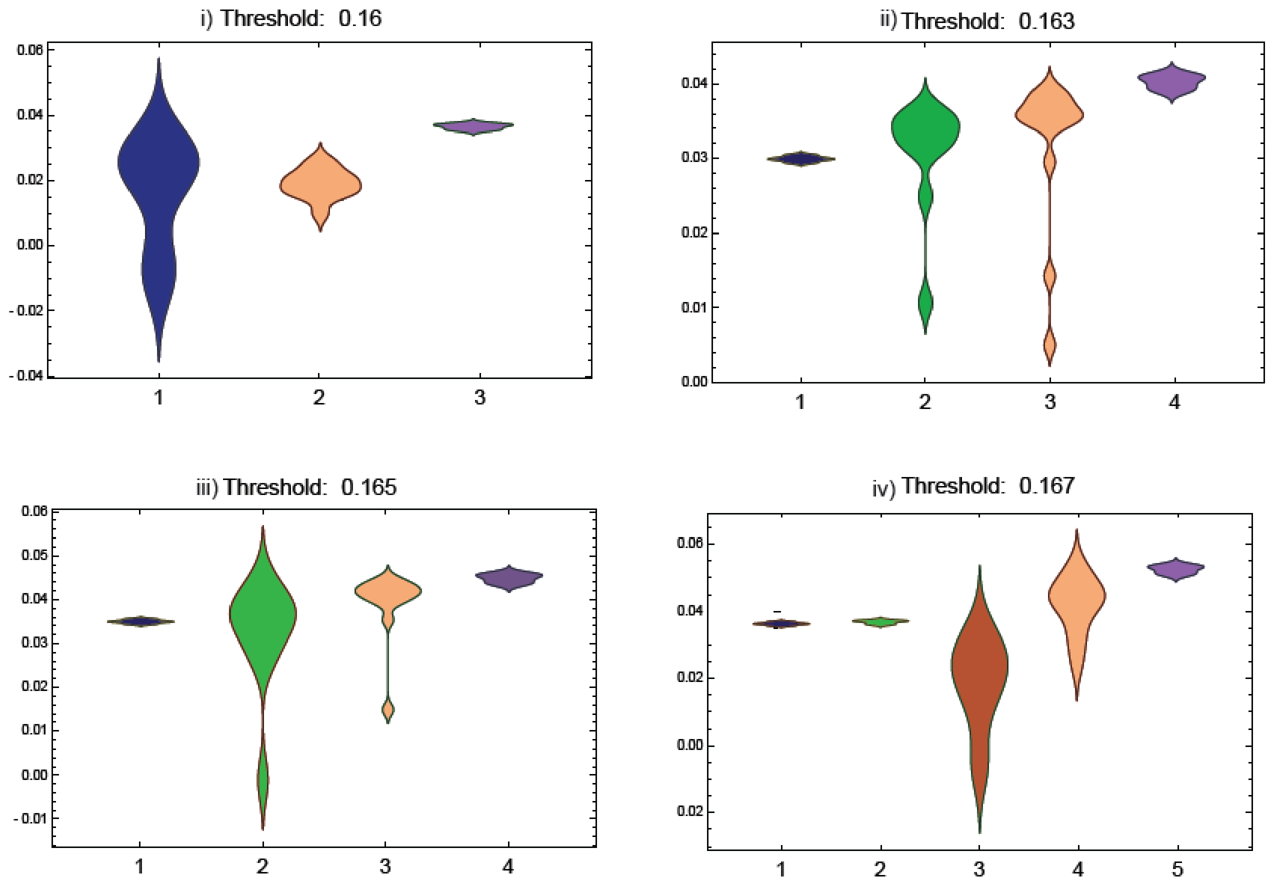

VIII) Genetic differentiation in peninsular India examined with the inclusion of a sample from Nagarjunsagar Srisailam Tiger Reserve (NSTR)

As discussed in the main text, our data suggests high differentiation between central and southern India. However, the only sample from NSTR, a population straddling the gap between central and southern India, could not be included in the analysis as it had very few reads remaining after filtering. From the final set of SNPs, the NSTR sample had only 88 SNPs. Admixture[9] analysis with NSTR included in the data still supports three clusters – NW, C and SI. NSTR derives nearly equal proportions of ancestry from C and SI (figure S12). However, as NSTR has a very large proportion of missing data, this is far from conclusive. We also repeated the analysis with only these 88 SNPs. In this case, K = 1 had the highest support, indicating that 88 SNPs have low power to detect structure.

Figure S12: Admixture plot with 39 individuals including NSTR at K = 3

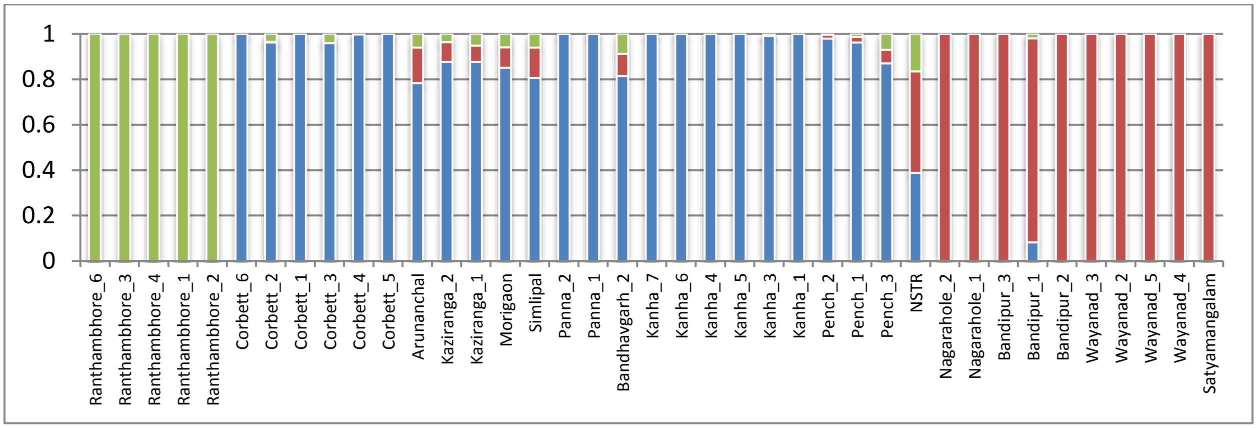

IX) Climate and vegetation based differentiation of tiger reserves

PCA was performed with climate (19 bioclim variables, 1 aridity index) and vegetation data (GlobCov land cover classes) for two sets of points – regular points across India, only points representing tiger reserves.

Figure S13: Contribution of variables to the PCA including all points. The proportion of variance explained by each axis is shown in brackets. Variables bio 1 – 19 correspond to the 19 climate variables from World BioClim data, Aridity_Ind measures aridity, and clipped_gl refers to vegetation from GlobCov. The colours indicate the contribution of each variable.

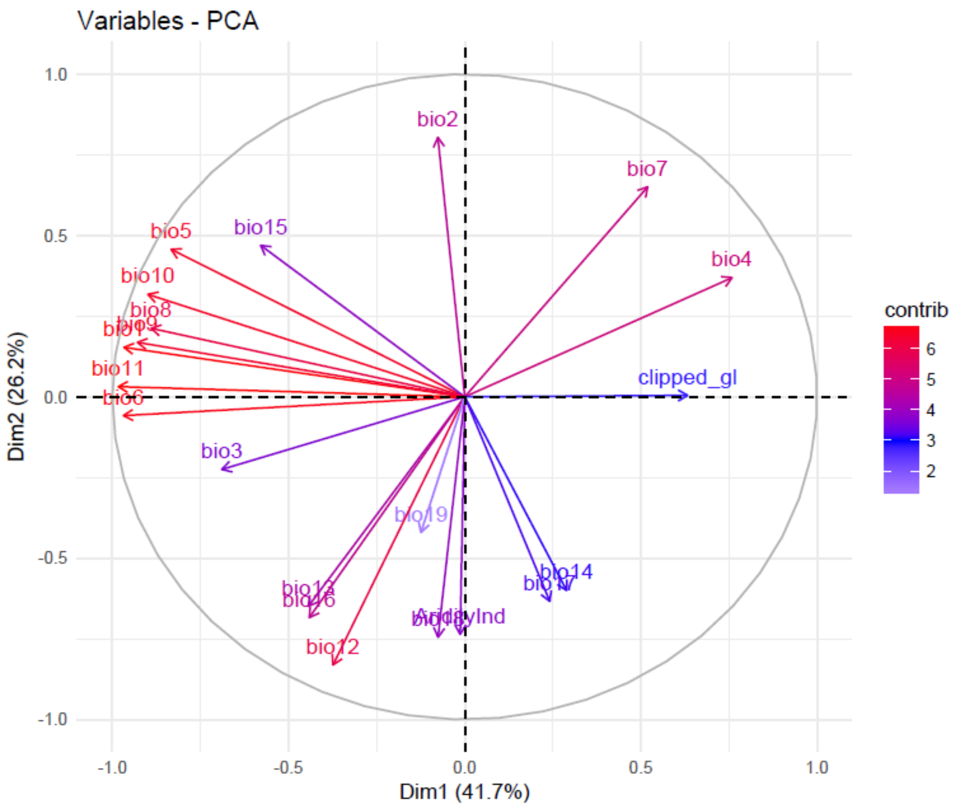

Figure S14: PCA of climate and vegetation data for all points. Red points represent regular points across India that fall outside protected areas. Names of tiger reserves are specified on the plot enclosed in rectangles.

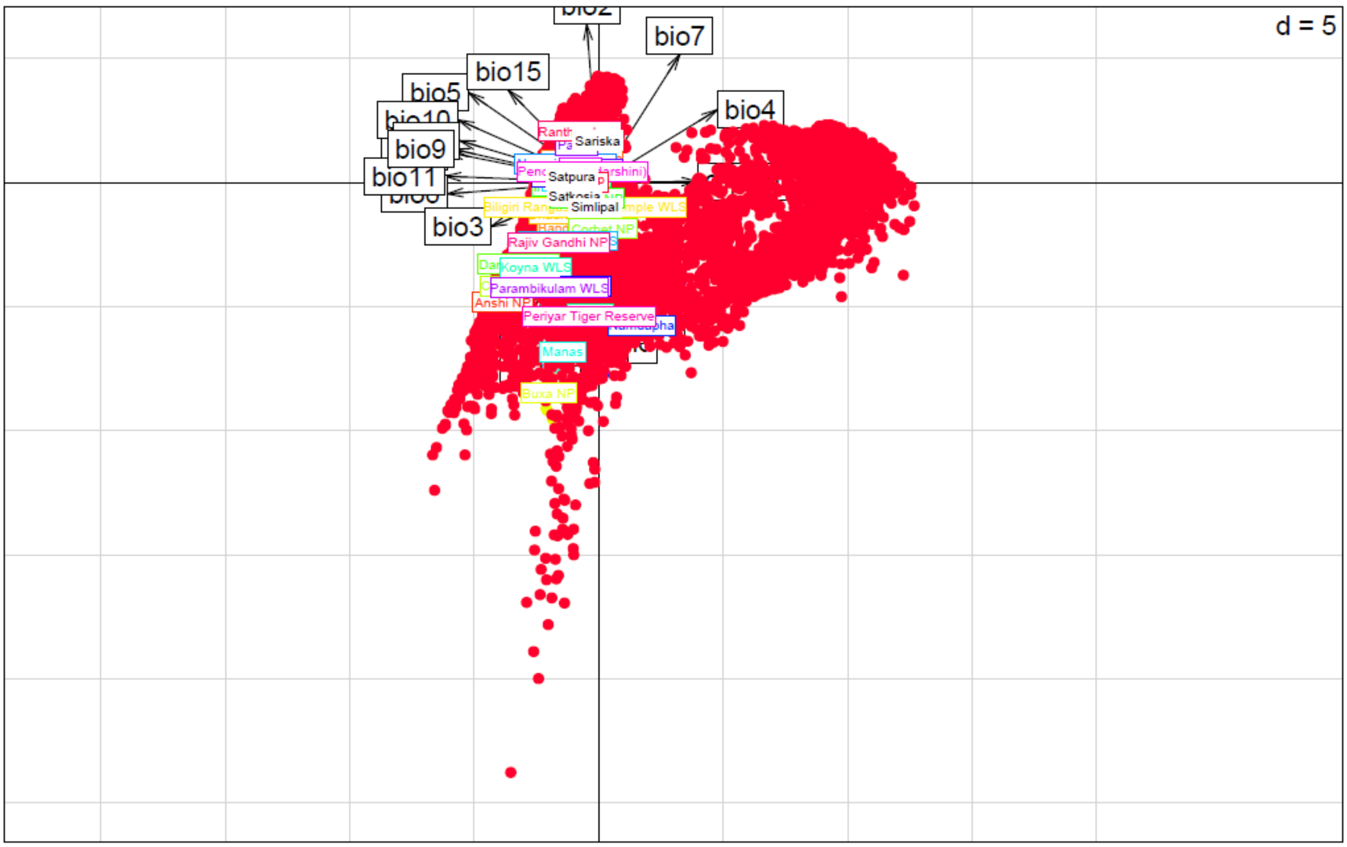

Figure S15: Contribution of variables to the PCA for points within tiger reserves. The proportion of variance explained by each axis is shown in brackets. Variables bio 1 – 19 correspond to the 19 climate variables from World BioClim data, Aridity_Ind measures aridity, and clipped_gl refers to vegetation from GlobCov. The colours indicate the contribution of each variable.

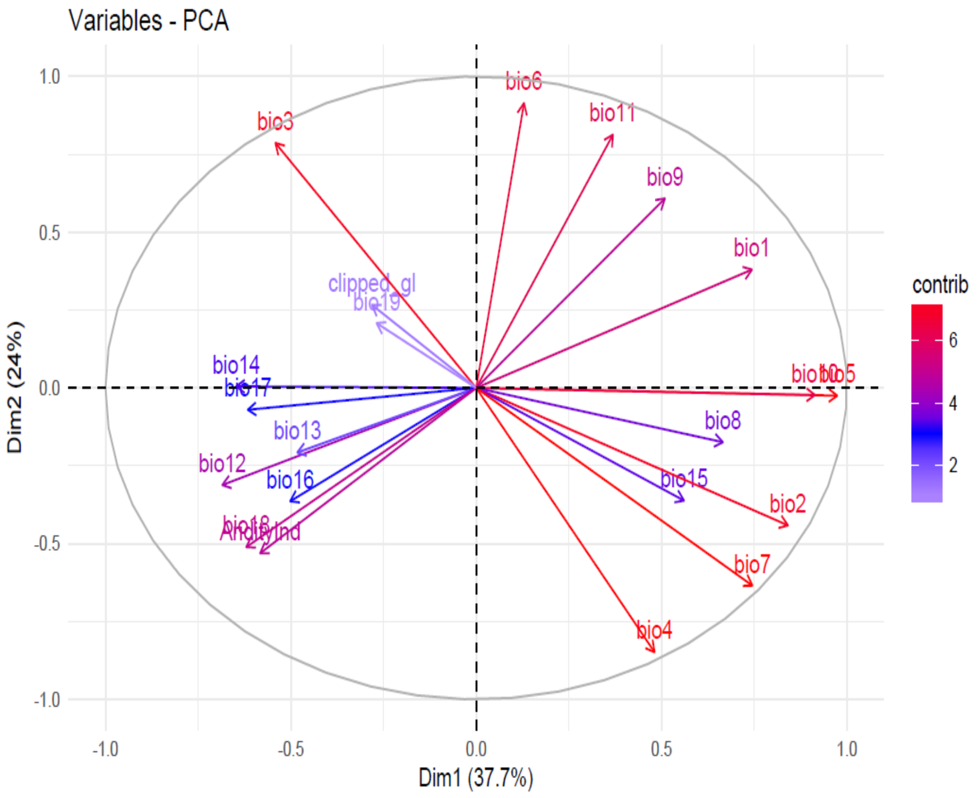

Figure S16: PCA of climate and vegetation data for points within tiger reserves. Names of tiger reserves are specified on the plot enclosed in rectangles.

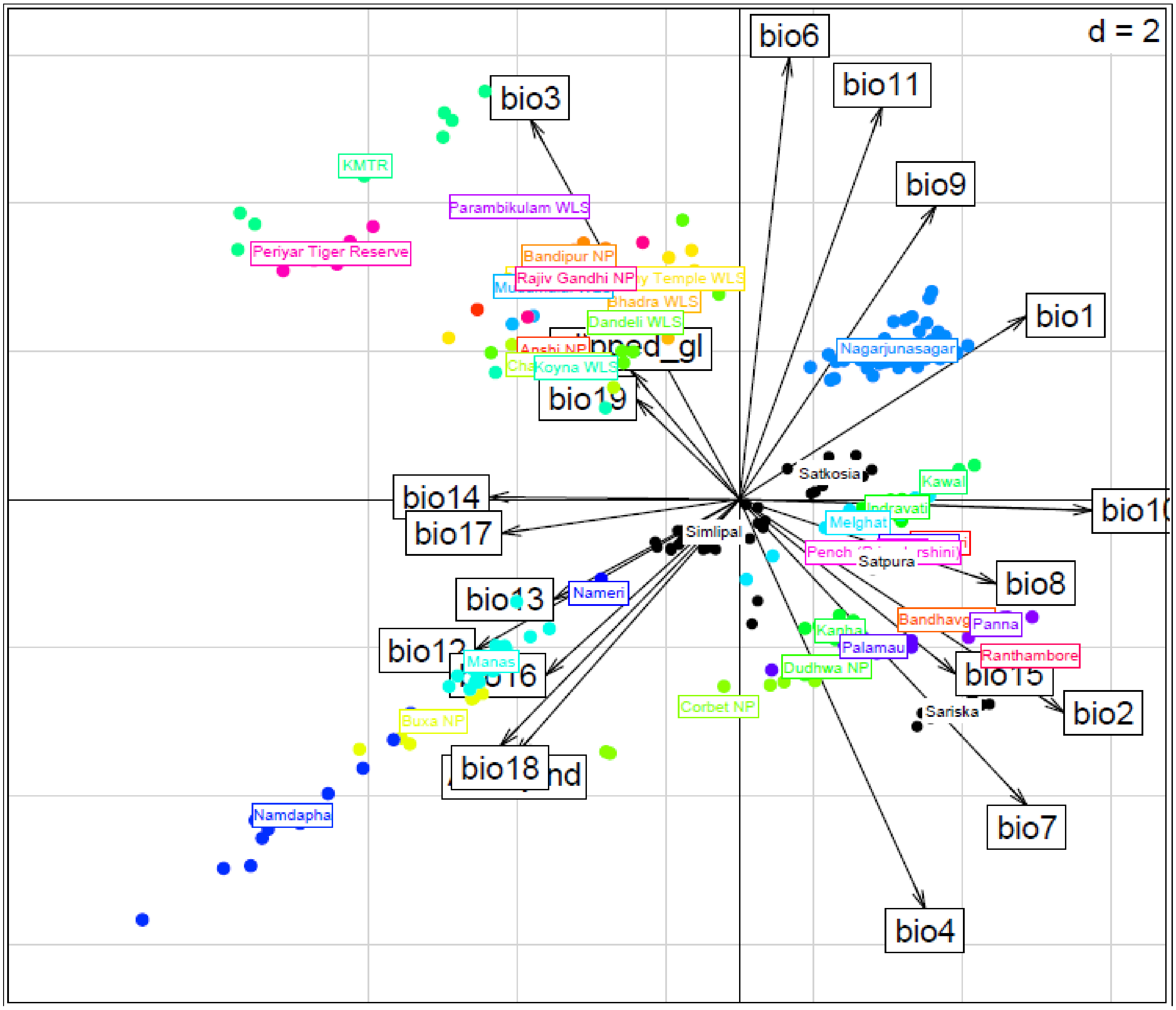

X) Population subdivision re-examined after removal of related individuals

As mentioned in the main text, it is difficult to sample wild tigers for tissue and our sampling is opportunistic. This has resulted in the inclusion of some related individuals in the data (Fig. S4). It also revealed the presence of a recapture which was then discarded from the data. While removing all related individuals for all analyses would have reduced the sample size quite a bit, we re-assessed population structure using just the unrelated individuals (p^ < 0.2) – 23 samples. For this reduced sample size, Admixture was unable to detect any structure. Therefore, we used FastStructure[10] for this purpose. We also analyzed the larger dataset with this algorithm to ensure that the results are comparable to that obtained from Admixture. Both the figures are shown below. For both analyses, FastStructure identified the model complexity that maximizes the marginal likelihood to be 4. For the larger dataset, K=3

was identified as the most likely value of K to describe the data, whereas 4 was the most likely value of K with FastStructure. However, the admixture proportions for each individual at K=4 were similar in both analysis.

Figure S17: Genetic clusters inferred at K=4 through FastStructure. All samples from the main study are included here.

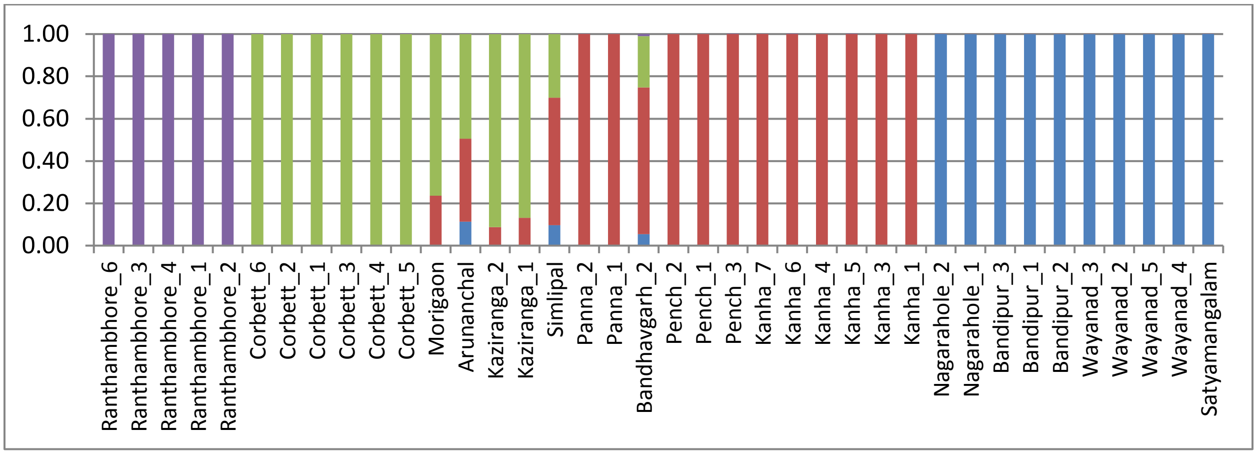

Figure S18: Genetic clusters inferred at K=4 through FastStructure for only unrelated individuals (p^ < 0.2).

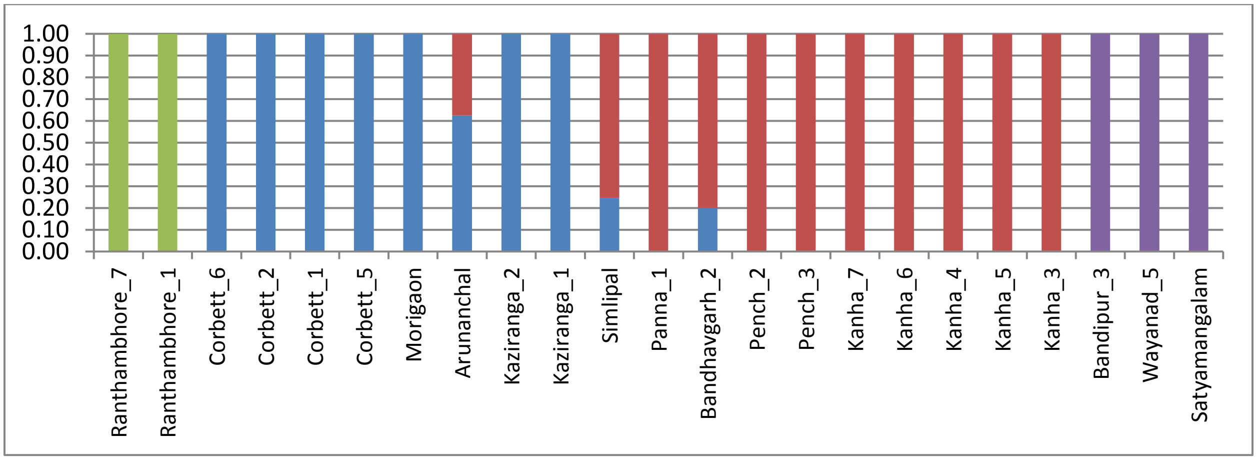

XII) Inference of demographic history using a site frequency spectrum-based method - DADI.

To derive the site frequency spectrum, SNP calls can directly be used. However, in the case of low coverage data such as ours, it is better to use methods that derive the site frequency spectrum (SFS) from genotype likelihoods[11]. Therefore, we first used the programs ANGSD[12] and realSFS to filter the bam files and obtain the site allele frequency likelihoods. These are used to estimate the EM optimized folded SFS. This was imported it into the *dadi*[13] environment. We began with single population models to test for population size

change in the past. However, none of the models could fit the data well and parameter estimates did not converge and would mostly hit against the specified bounds. A possible reason could be low sample sizes of the populations in question. A simulation-based study suggests that detecting recent bottlenecks would require more than 10 individuals per population[14]. It is possible that given the very recent bottleneck experienced by tigers, it would require a larger dataset than ours to examine the demographic history of these populations. Below is the fit of the data to a 2 epoch model from our *dadi* runs (Fig. S19). As can be seen from the figure, the sfs shows a huge deficit of low frequency alleles. The fit of the data to other one population models was similar.

Figure S19: Model fit of the data to a 2-epoch model of demographic history. The upper panel shows the data (blue) and the model (red) and the lower panel shows the residuals. The X axis depicts the number of chromosomes and the Y axis depicts the frequency.

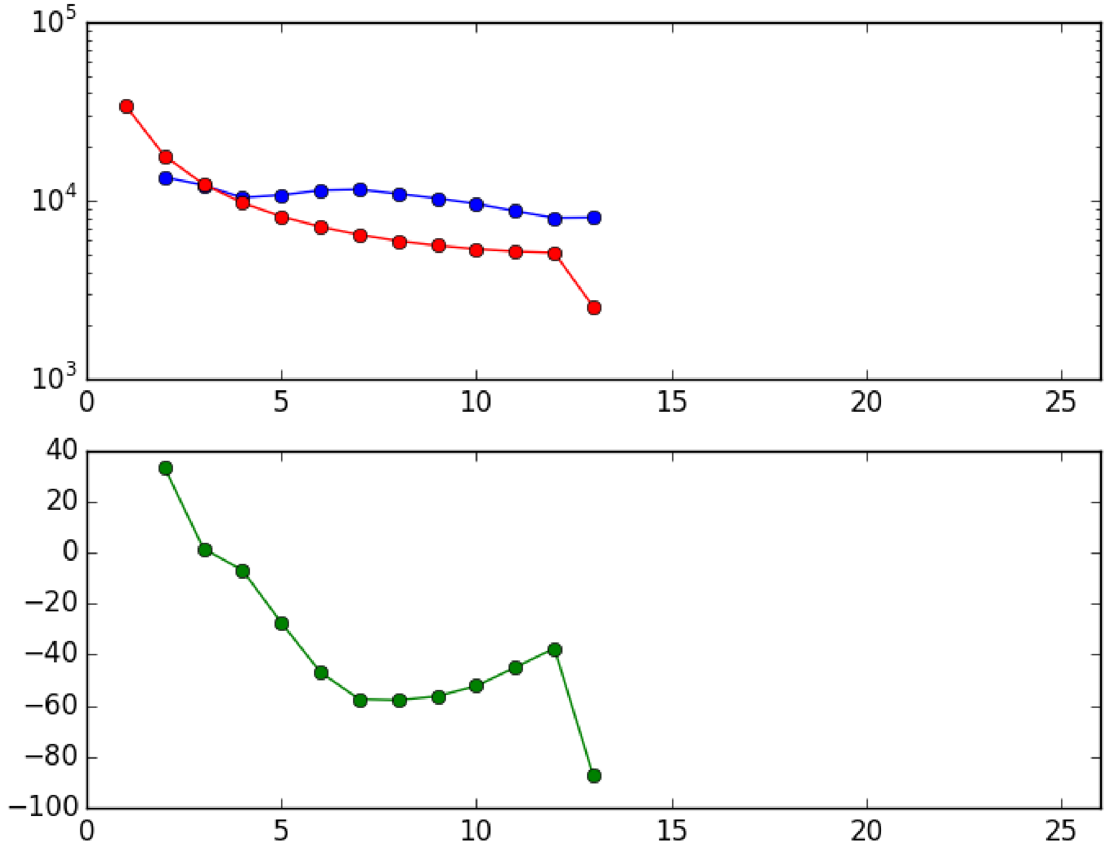

Bibliography:

1. Catchen, J., Hohenlohe, P. a, Bassham, S., Amores, A. & Cresko, W. a. Stacks: an analysis tool set for population genomics. *Mol. Ecol.* **22,** 3124–40 (2013).

2. Garrison, E. & Marth, G. Haplotype-based variant detection from short-read sequencing. *bioRxiv* **1207.3907,** (2013).

3. Quinlan, A. R. & Hall, I. M. BEDTools: A flexible suite of utilities for comparing genomic features. *Bioinformatics* **26,** 841–842 (2010).

4. Purcell, S. *et al.* PLINK: a tool set for whole-genome association and population-based linkage analyses. *Am. J. Hum. Genet.* **81,** 559–75 (2007).

5. Excoffier, L., Laval, G. & Schneider, S. Arlequin (version 3.0): an integrated software package for population genetics data analysis. *Evol. Bioinform. Online* **1,** 47–50 (2005).

6. Jombart, T. Adegenet: A R package for the multivariate analysis of genetic markers. *Bioinformatics* **24,** 1403–1405 (2008).

7. Foll, M. & Gaggiotti, O. A genome-scan method to identify selected loci appropriate for both dominant and codominant markers: A Bayesian perspective. *Genetics* **180,** 977–993 (2008).

8. Greenbaum, G., Templeton, A. R. & Bar-David, S. Inference and analysis of population structure using genetic data and network theory. *Genetics* **202,** 1299-1312; DOI: 10.1534/genetics.115.182626 (2016).

9. Alexander, D. H. & Novembre, J. Fast Model-Based Estimation of Ancestry in Unrelated Individuals. *Genome Research* **19**, 1655-1664; DOI:10.1101/gr.094052.109 (2009).

10. Raj, A., Stephens, M. & Pritchard, J. K. FastSTRUCTURE: Variational inference of population structure in large SNP data sets. *Genetics* **197,** 573–589 (2014).

11. Han, E., Sinsheimer, J. S. & Novembre, J. Characterizing bias in population genetic inferences from low-coverage sequencing data. *Mol. Biol. Evol.* **31,** 723–735 (2014).

12. Korneliussen, T. S. T. *et al.* ANGSD: Analysis of Next Generation Sequencing Data. *BMC Bioinformatics* **15,** 1–13 (2014).

13. Gutenkunst, R. N., Hernandez, R. D., Williamson, S. H. & Bustamante, C. D. Inferring the joint demographic history of multiple populations from multidimensional SNP frequency data. *PLoS Genet.* **5,** (2009).

14. Robinson, J. D., Coffman, A. J., Hickerson, M. J. & Gutenkunst, R. N. Sampling strategies for frequency spectrum-based population genomic inference. *BMC Evol. Biol.* **14,** 254 (2014).
